# Supplementary material for: Choice between implants in knee replacement: protocol for a Bayesian network meta-analysis, analysis of joint registries and economic decision model to determine the effectiveness and cost-effectiveness of knee implants for NHS patients—The KNee Implant Prostheses Study (KNIPS)
Source: BMJ Open. 2021 Jan 6;11(1):e040205. doi: 10.1136/bmjopen-2020-040205 (PMC7789438; doi:10.1136/bmjopen-2020-040205)

Supplementary material. Economic decision models

Model structure

Potential model structures were developed by the research team with the help of surgeons, patients, and searches for previous economic models. We will start by applying a Markov decision model with tunnel states to reflect the three different time periods at risk, as depicted in Figure 1.

Figure 1. A Markov model using tunnel states to model outcomes after total knee replacement (`A').*

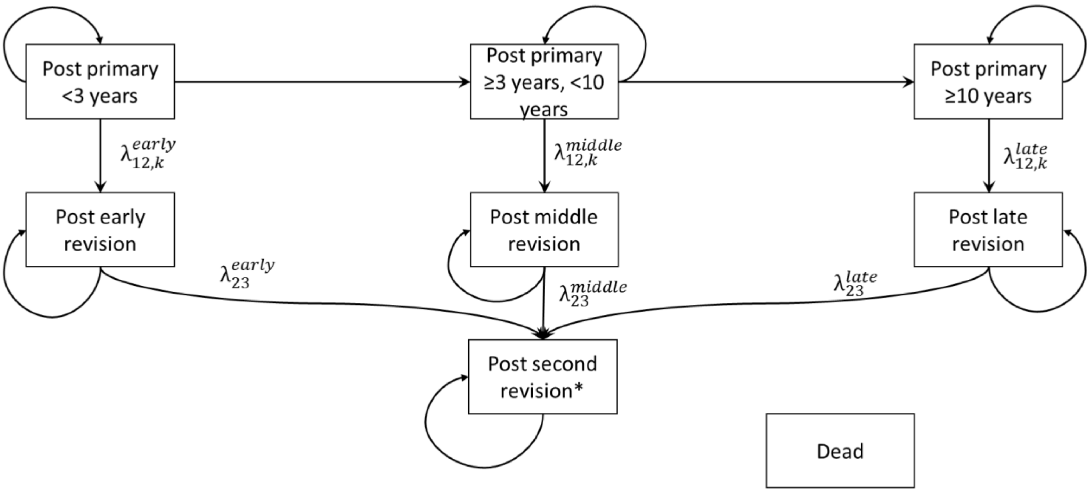

*Indices of transition rates λ_{ij} correspond to sets of states i and j: "Post TKR" ($i = 1$), Post revision ($i = 2$), and post second revision ($i = 3$).

States are divided into three groups corresponding to post primary (set 1), post revision (set 2), and post second revision (set 3) surgery. This model splits revisions into three time periods: up to 3 years post primary surgery ("early"), between 3- and 10-years post-primary ("middle"), and 10 or more years post primary surgery ("late"). This division applies to the post primary and post revision sets of states.

The three post primary states have three log hazard rates of revision $\lambda_{12,k}^{early}$, $\lambda_{12,k}^{middle}$, and $\lambda_{12,k}^{late}$, for implant k, such as:

$$\text{Equation 1. } \lambda_{12,k}^{period} = \alpha_{12}^{period} + d_{12,k}^{period}$$

In Equation 1, period is "early", "middle", or "late" first revision.

α_{12}^{period} is the log hazard of 1st revision for the baseline implant and informed by National Joint Registry (NJR) or Swedish Knee Arthroplasty Register (SKAR) data, which is constant over time within each 'period'. $d_{12,k}^{period}$ is the log hazard ratio of 1st revision for implant k

relative to the baseline and it is informed by the results of the network meta-analysis (NMA), combined with NJR or SKAR data. If NMA estimates are too sparse or uncertain, will use observational estimates from registries only.

These time-independent log rates are converted to annual probabilities for the 1-year cycle length using the exponential distribution

Equation 2. $P_{12,k}^{period} = 1 - \exp\left(-\exp\left(\lambda_{12,k}^{period}\right)\right)$

The rates of second revision are assumed to depend on whether the first revision occurred in the "early", "middle", or "late" first revision period, with constant log hazards λ_{23}^{early}, λ_{23}^{middle}, and λ_{23}^{late}. These are again converted to probabilities using an exponential distribution

Equation 3. $P_{23}^{period} = 1 - \exp\left(-\exp\left(\lambda_{23}^{period}\right)\right)$

The log rates of second revision will be estimated using NJR and SKAR data and will be assumed to be the same for all implants (hence no index k); note that they are different to the log rates of first revision.

The annual probability of death $P_M(u)$ will be assumed independent of current state or implant. It will depend on current time u as it will increase with age as in the Office of National Statistics life tables. Dependence of all log hazards of 1st and 2nd revision on age and gender will be managed through stratification into male/female and age bands (e.g. <55, 55-64, 65-74, 75+ years old).

We will also explore using an individual level continuous time semi-Markov multistate model, which simulates individual patient paths in continuous time.

A continuous model allows the hazard rate and hazard rate ratios of first revision to vary continuously, giving a log transition rate of 1st revision function $\lambda_{12,k}(u, x_i)$ that depends on current time u and, possibly, on patient characteristics x_i. The constant hazards over the early, middle, and late periods in the cohort Markov model are an approximation to this function. The log rate $\lambda_{12,k}(u, x_i)$ is generated using baseline implant hazards $\alpha_{12}(u, x_i)$ (i.e. $k = 1$) from NJR/SKAR and hazard ratios $d_{12,k}(u, x_i)$ from NJR/SKAR and the NMA for other implants k:

Equation 4. Individual patient semi-Markov : $\lambda_{12,k}(u, x_i) = \alpha_{12}(u, x_i) + d_{12,k}(u, x_i)$

Unlike the cohort Markov model the log rates of revision on the baseline implant $\alpha_{12}(u, x_i)$ and log hazard ratios $d_{12,k}(u, x_i)$ for implant k are now functions (rather than three constants).

The hazard of second revision $\lambda_{23}(u, t_{12,i})$ will depend on the time taken by individual patient i to experience first revision $t_{12,i}$ (i.e. time to transition to from 1st to 2nd state). Mortality is modelled as a log hazard of death $\lambda_M(u, x_i)$ which is assumed independent of current state or implant but may depend on patient characteristics x_i (e.g. age and gender)

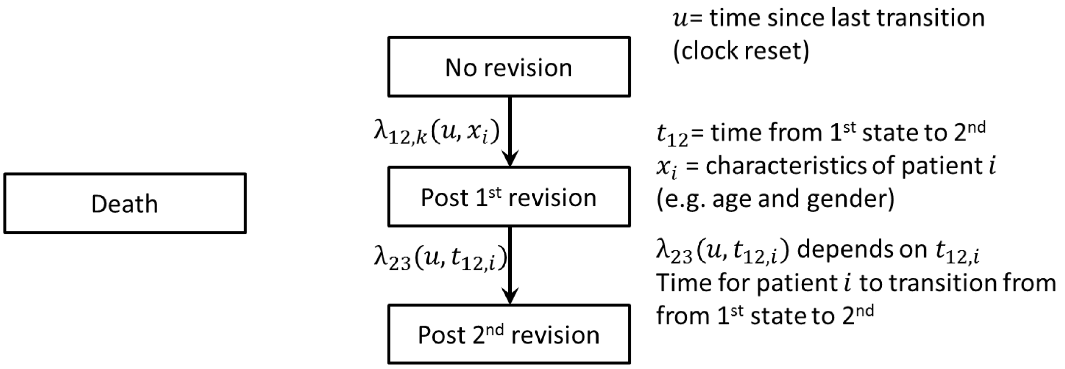

Supplement: Supplementary data [file bmjopen-2020-040205supp001.pdf]
