# Supplementary material for: Choice between implants in knee replacement: protocol for a Bayesian network meta-analysis, analysis of joint registries and economic decision model to determine the effectiveness and cost-effectiveness of knee implants for NHS patients—The KNee Implant Prostheses Study (KNIPS)
Source: BMJ Open. 2021 Jan 6;11(1):e040205. doi: 10.1136/bmjopen-2020-040205 (PMC7789438; doi:10.1136/bmjopen-2020-040205)
Supplement: Author's manuscript [file bmjopen-2020-040205.draft_revisions.pdf]

BMJ Open

BMJ Open is committed to open peer review. As part of this commitment we make the peer review history of every article we publish publicly available.

When an article is published we post the peer reviewers' comments and the authors' responses online. We also post the versions of the paper that were used during peer review. These are the versions that the peer review comments apply to.

The versions of the paper that follow are the versions that were submitted during the peer review process. They are not the versions of record or the final published versions. They should not be cited or distributed as the published version of this manuscript.

BMJ Open is an open access journal and the full, final, typeset and author-corrected version of record of the manuscript is available on our site with no access controls, subscription charges or pay-per-view fees (http://bmjopen.bmj.com).

If you have any questions on BMJ Open's open peer review process please email info.bmjopen@bmj.com

BMJ Open

The choice between implants in knee replacement: Protocol for a Bayesian network meta-analysis, analysis of joint registries and economic decision model to determine the effectiveness and cost-effectiveness of knee implants for NHS patients. The KNee Implant Prostheses Study (KNIPS)

Journal:	*BMJ Open*
Manuscript ID	bmjopen-2020-040205
Article Type:	Protocol
Date Submitted by the Author:	07-May-2020
Complete List of Authors:	Marques, Elsa; Bristol Medical School, Musculoskeletal Research Unit<br>Dennis, Jane; Bristol Medical School, Musculoskeletal Research Unit<br>Beswick, Andrew; Bristol Medical School, Musculoskeletal Research Unit<br>Higgins, Julian; Bristol Medical School, Population Health Sciences<br>Thom, H; Bristol Medical School, Population Health Sciences<br>Welton, Nicky; Bristol Medical School, Population Health Sciences<br>Burston, Amanda; Bristol Medical School, Musculoskeletal Research Unit<br>Hunt, Linda; Bristol Medical School, Musculoskeletal Research Unit<br>Whitehouse, Michael; Bristol Medical School, Musculoskeletal Research Unit<br>Blom, AW; Bristol Medical School, Musculoskeletal Research Unit
Keywords:	Knee < ORTHOPAEDIC & TRAUMA SURGERY, Adult orthopaedics < ORTHOPAEDIC & TRAUMA SURGERY, STATISTICS & RESEARCH METHODS

SCHOLARONE™
Manuscripts

I, the Submitting Author has the right to grant and does grant on behalf of all authors of the Work (as defined in the below author licence), an exclusive licence and/or a non-exclusive licence for contributions from authors who are: i) UK Crown employees; ii) where BMJ has agreed a CC-BY licence shall apply, and/or iii) in accordance with the terms applicable for US Federal Government officers or employees acting as part of their official duties; on a worldwide, perpetual, irrevocable, royalty-free basis to BMJ Publishing Group Ltd ("BMJ") its licensees and where the relevant Journal is co-owned by BMJ to the co-owners of the Journal, to publish the Work in this journal and any other BMJ products and to exploit all rights, as set out in our licence.

The Submitting Author accepts and understands that any supply made under these terms is made by BMJ to the Submitting Author unless you are acting as an employee on behalf of your employer or a postgraduate student of an affiliated institution which is paying any applicable article publishing charge ("APC") for Open Access articles. Where the Submitting Author wishes to make the Work available on an Open Access basis (and intends to pay the relevant APC), the terms of reuse of such Open Access shall be governed by a Creative Commons licence – details of these licences and which Creative Commons licence will apply to this Work are set out in our licence referred to above.

Other than as permitted in any relevant BMJ Author's Self Archiving Policies, I confirm this Work has not been accepted for publication elsewhere, is not being considered for publication elsewhere and does not duplicate material already published. I confirm all authors consent to publication of this Work and authorise the granting of this licence.

For peer review only

The choice between implants in knee replacement: Protocol for a Bayesian network meta-analysis, analysis of joint registries and economic decision model to determine the effectiveness and cost-effectiveness of knee implants for NHS patients. The KNee Implant Prostheses Study (KNIPS)

Elsa M R Marques[1]

Jane Dennis[1]

Andrew D Beswick[1]

Julian PT Higgins[2 3]

Howard Thom[2 3]

Nicky J Welton[2 3]

Amanda Burston[1]

Linda P Hunt[1]

Michael R Whitehouse[1 3]

Ashley W Blom[1 3]

[1] Musculoskeletal Research Unit, Translational Health Sciences, Bristol Medical School, University of Bristol

[2] Population Health Sciences, Bristol Medical School, University of Bristol, Bristol

[3]National Institute for Health Research Bristol Biomedical Research Centre, University Hospitals Bristol NHS Foundation Trust and University of Bristol

e.marques@bristol.ac.uk

jane.dennis@bristol.ac.uk

andy.beswick@bristol.ac.uk

julian.higgins@bristol.ac.uk

howard.thom@bristol.ac.uk

nicky.welton@bristol.ac.uk

amanda.burston@bristol.ac.uk

l.p.hunt@bristol.ac.uk

For peer review only

michael.whitehouse@bristol.ac.uk

ashley.blom@bristol.ac.uk

Corresponding author: Andrew D Beswick

Musculoskeletal Research Unit

Translational Health Sciences

Bristol Medical School

University of Bristol

Learning and Research Building, Level 1

Southmead Hospital

Bristol

BS10 5NB

andy.beswick@bristol.ac.uk

Tel 01174147870

Word count: 5150

Keywords:

Arthroplasty, Replacement, Knee

Unicompartmental knee replacement

Network meta-analysis

Prostheses and implants

Cost benefit analysis

Registries

For peer review only

Abstract

Introduction

Knee replacements are highly successful for many people, but if a knee replacement fails, revision surgery is generally required. Surgeons and patients may choose from a range of implant components and combinations which make up knee replacement constructs, all with potential implications for how long a knee replacement will last. To inform surgeon and patient decisions, a comprehensive synthesis of data from randomised controlled trials is needed to evaluate the effects of different knee replacement constructs on survival. Due to limited follow-up in trials, joint registry analyses are also needed to assess their long-term survival. Finally, economic modelling can identify cost-effective knee replacement constructs for different patient groups.

Methods and analysis

In this protocol we describe systematic reviews and network meta-analyses to synthesise evidence on the effectiveness of knee replacement constructs used in total and unicompartmental knee replacement, and analyses of two national joint registries to assess long-term outcomes. Knee replacement constructs will be defined by bearing materials and mobility, constraint, fixation and patella resurfacing. For men and women in different age groups, we will compare the lifetime cost-effectiveness of different knee replacement constructs.

Ethics and dissemination

Systematic reviews are secondary analyses of published data with no ethical approval required. We will design a common joint registry analysis plan and provide joint registry representatives with information for submission to research or ethics committees. The project has been assessed by the NHS REC committee and does not require ethical review.

Study findings will be disseminated to clinicians, researchers and administrators through open access articles, presentations and websites. Specific UK-based groups will be informed of results including NIHR and NICE, as well as international orthopaedic associations and charities. Effective dissemination to patients will be guided by our patient-public involvement group and will include written lay summaries and infographics.

PROSPERO registration numbers: CRD42019134059; CRD42019138015

Article summary

For peer review only - http://bmjopen.bmj.com/site/about/guidelines.xhtml

Strengths and limitations of the study

- Bringing evidence from multiple RCTs together in systematic reviews and meta-analysis with thorough assessments of risk of bias and confidence in the evidence ensures that health professionals have the information they need to deliver a high-quality patient experience with safe, clinically effective and cost-effective treatments.
- Many RCTs of knee replacement constructs report radiographic outcomes, which do not necessarily require large sample sizes and long-term follow up; thus, a limitation of the systematic review elements of our project is the likely inclusion of small RCTs with short follow up.
- The joint registries that we will analyse include large numbers of patients with information on knee replacement constructs used and patient demographics such as age and sex, to enable adjustment for potential confounders.
- In registries, treatment choices are made for individual patients and uncontrolled confounding factors may influence outcomes.
- Exploring the effectiveness of knee replacement constructs in both RCTs and registries, and linking this information to implant and healthcare costs, will allow us to compare the lifetime cost-effectiveness of different knee replacement constructs.

For peer review only

Introduction

In 2018, more than 100,000 primary knee replacements were performed in the UK(1,2) and over 15,400 in Sweden.(3) About 96% of operations are to treat end-stage osteoarthritis, with the majority in women (57%) at a mean age of 69 years.(1) For many people, knee replacements are highly successful and can last a person's lifetime.(4) However, when a knee replacement fails, most commonly due to loosening or wear, people may require revision surgery. Revision surgery is more complex for clinicians to perform, is difficult for patients to recover from, is associated with further complications and need for re-revisions, and is costly.

Description of the intervention

The knee joint consists of three compartments: the medial femorotibial, the lateral femorotibial and the patellofemoral. Depending on which compartments are affected by osteoarthritis, a surgeon may perform a total knee replacement (TKR) or a unicompartmental knee replacement (UKR). In TKR, both the medial and lateral compartments are replaced as well as the trochlea (groove) on the front of the femur. The patella that articulates with this may be resurfaced or not. Some surgeons favour UKR on the basis of radiographic evidence of osteoarthritis affecting a single medial or lateral compartment with estimates of patient eligibility for UKR as high as 48% of all people receiving knee replacement,(5) although actual rates of utilisation are around 10%.(1,3) Compared with TKR, UKR surgery requires a less invasive procedure, retains more bone and native ligaments, has a shorter duration, is quicker for patients to recover from, and is cheaper for the NHS. However, with time, osteoarthritis can develop in the other compartments and a patient with a UKR is more than twice as likely to undergo revision than a patient who has had a TKR.(1) Revision of UKR to TKR can be complex, and may include treatment of tibial bone defects.(6) Thus, many surgeons favour TKR as the first treatment for severe knee osteoarthritis. In the National Joint Registry for England, Wales, Northern Ireland and the Isle of Man (NJR), 11% of knee replacements performed in 2018 were UKR.(1) In 2018 in the Swedish Knee Arthroplasty Register (SKAR), 9.6% of knee replacements were UKR.(3)

In both TKR and UKR, the remaining cartilage and some bone in the affected portion of the joint is removed and replaced, typically with implants made of metal fixed to the bone and polyethylene bearing surfaces between the metal implants or affixed directly to the bone on the opposite side of the joint. Stability of the knee relies to a large degree on the ligaments in and around the knee. In UKR, these are retained, in TKR, some of them are removed and their function compensated for by the implants used.

For peer review only - http://bmjopen.bmj.com/site/about/guidelines.xhtml

TKR construct options (Table 1)

Surgeons and patients may choose from a range of implant components and combinations which make up knee replacement constructs, all with potential implications for how long a knee replacement will last.

In a modular TKR construct, the polyethylene liner between the metal femoral and tibial components can be “fixed” to the tibial component or “mobile” with movement of the liner permitted on the tibial component. In a “monobloc” implant, the top of the tibia is replaced with a one-piece polyethylene implant that is fixed directly to the bone.

TKR constructs can accommodate keeping the posterior cruciate ligament (cruciate retaining) or not (cruciate sacrificing or posterior stabilised). Depending on the condition of the patella and the opinion of the surgeon, the patella can be resurfaced with a polyethylene or metal-backed polyethylene implant.

Fixation of the femoral and tibial components to their respective bones can be done with or without the use of cement. In a hybrid TKR, one of the components is fixed with cement whilst the other is uncemented.

UKR construct options (Table 1)

Depending on which compartment is affected and replaced, UKRs can be medial UKR, lateral UKR or patellofemoral UKR. As with TKR, component options are available to surgeons performing a UKR. The polyethylene liner may be fixed or mobile and implants can be fixed to bone with cement, without cement (uncemented) or using a combination (hybrid). In patellofemoral UKR, the back of the patella and trochlea of the femur are resurfaced with polyethylene and metal respectively. In rare situations, surgeons may perform multiple UKRs of different compartments at the same time.(7)

Effectiveness and cost-effectiveness of knee replacement constructs: existing knowledge

To assess the relevance of our proposed research, we performed a scoping literature search in January 2020 to identify existing network meta-analyses relating to knee replacement. Most of the 33 studies we identified considered drug treatments. Two systematic reviews and one protocol described the application of network meta-analysis to knee replacement constructs. One study compared resurfacing of the patella with no resurfacing and, to complete the network, with patella denervation.(8) While the authors concluded that patella resurfacing was associated with a lower rate of re-operation than not resurfacing the patella and with no benefit for pain or surgeon-assessed scores, there was no attempt to interpret

For peer review only - http://bmjopen.bmj.com/site/about/guidelines.xhtml

results in the context of assessed risk of bias of included studies. Another systematic review of different surgeries for the treatment of osteoarthritis with network meta-analysis only included cohort studies and did not assess their risk of bias.(9) A third Cochrane review protocol describing a network meta-analysis to compare diverse surgical and medical therapies in TKR(10) was withdrawn in September 2019.(11)

In a second contemporaneous search of the same databases for systematic reviews, we identified six that explicitly considered the cost-effectiveness of different knee replacement constructs. The potential value of patella resurfacing was evaluated in one systematic review.(12) Over five years, the authors concluded that there was a small cost-saving when the patella was resurfaced. However, there was no consideration of risk of bias. In one systematic review, the authors concluded that the benefit of UKR compared with TKR is dependent on the economic perspective chosen, patient characteristics and the timing of outcome.(13) In three reviews, authors concluded that UKR may be a cost-effective outcome in older patients but that this was uncertain for younger patients due to lower survivorship associated with UKR constructs.(14-16) In another systematic review comparing UKR, TKR and other procedures, results presented narratively were equivocal.(17)

A further search for economic analyses and registry studies identified 13 potentially relevant studies. Registry analyses covered bearing surfaces,(18-20) monobloc tibial components,(21) patella resurfacing,(22) mobile and high flexion designs,(19) fixation,(23) patellofemoral replacement,(24) and UKR.(16,25-27) We did not find any registry studies comparable to our study comparing diverse features of knee replacement constructs. The authors of one study presented a cost-effectiveness model based on five common brands of TKR implants up to 10 years post-primary surgery.(28) Each brand studied represented a construct with cemented unconstrained components with fixed bearings, in contrast with our approach, which focuses on different constructs.

A search of PROSPERO in January 2020 identified no network meta-analyses in knee replacement comparing knee replacement constructs comparable to our planned study.

Objectives

The objectives of our study are to synthesise evidence from randomised controlled trials (RCTs), registries, and studies reporting quality of life and cost information, to identify:

1. the most effective and cost-effective TKR constructs for patients of different age and sex profiles
2. the most effective and cost-effective knee replacement constructs for patients of different age and sex profiles eligible for both TKR and UKR

For peer review only - http://bmjopen.bmj.com/site/about/guidelines.xhtml

We will achieve these objectives by conducting two systematic reviews to identify RCT evidence for each question, analysis of registries from the UK and Sweden to estimate long-term revision and mortality rates, and development of a cost-effectiveness model for each question.

The primary outcome in both systematic reviews will be revision rate and timing of revision which are key markers of construct effectiveness. Secondary outcomes will be patient-reported pain and function, and surgeon-assessed outcomes.

As the implants that make up a construct can vary in TKR and UKR, network meta-analysis with direct and indirect comparisons is an appropriate method for synthesis of data from RCTs. We will develop networks of evidence for revision (and other outcomes) at different time periods after primary TKR and UKR. These will include an initial period post primary surgery where the risk of first revision is high, a medium-term period with a lower risk of first revision and a late revision period, where risk of first revision increases.

Recognising the short follow-up in many orthopaedic RCTs, we will also analyse data on outcomes after different knee replacement constructs from two joint registries.

Outcome data from RCTs and registries, together with evidence on quality of life and construct and health service costs, will be used to inform economic decision models which will rank and estimate the cost-effectiveness of knee constructs for patients of different sex and age profiles.

Methods

Patient and public involvement

This proposal was developed in collaboration with the University of Bristol Musculoskeletal Research Unit patient and public involvement group. The “Patient Experience Partnership in Research” (PEP-R) group comprises nine members, most of whom have had joint replacement. Meetings are facilitated by a dedicated coordinator who works in partnership with researchers to provide patient and public input into research. We met with PEP-R on two occasions for input into the development of the research proposal in 2017 and 2018, and at the start of the project in 2019. Patients told us that they were not informed about knee implant options when discussing their surgery with the consultant. PEP-R will provide ongoing support throughout the study and appropriate funding is in place. Anticipated contributions will include advice on dissemination of results, particularly plain language summaries.

Systematic reviews and meta-analyses

For peer review only - http://bmjopen.bmj.com/site/about/guidelines.xhtml

The systematic reviews are registered with PROSPERO (CRD42019134059 and CRD42019138015) and a Preferred Reporting Items for Systematic Reviews and Meta-Analyses Protocols (PRISMA-P) statement(29) is provided. The research questions are formulated according to PICO(30) and review conduct will be based on methods described in the Cochrane Handbook for Systematic Reviews of Interventions.(31) Reporting will adhere to the Preferred Reporting Items for Systematic Reviews and Meta-Analyses (PRISMA) guidelines(32) and the PRISMA extension statement for reporting of systematic reviews incorporating network meta-analyses.(33)

Systematic review 1: TKR

Eligibility criteria

Types of studies

We will include RCTs.

Participants

Eligible patients will be receiving elective primary TKR (unilateral or bilateral) and aged 18 years or older. There will be a diagnosis of osteoarthritis in 50% or more of the study population.

Interventions and comparators

We will include comparisons of knee replacement constructs as summarised in Table 1. Each construct and comparator will have a combination defined by bearing mobility, constraint, fixation, bearing materials and patella resurfacing. In theory this could be 240 different combinations, but many combinations are not feasible or desirable.

The reference construct within the network meta-analysis will be the most widely used TKR construct which has cemented, cruciate retaining, fixed bearing implant components with metal on polyethylene bearing materials.

Types of outcome measures

The primary outcome of the systematic reviews will be first revision surgery after primary TKR and UKR at any time from primary surgery. We will collect data at each time point reported in articles, and extract data from published Kaplan-Meier curves if available.(34)

Secondary outcomes will include: further revision surgeries; deaths; reason for revision; patient-reported outcomes such as the Oxford Knee Score (OKS)(35) and Western Ontario and McMaster Universities Arthritis Index (WOMAC);(36) clinician-assessed measures

For peer review only - http://bmjopen.bmj.com/site/about/guidelines.xhtml

including the American Knee Society Score (KSS)(37) and Hospital for Special Surgery (HSS) score;(38) and quality of life indices such as the EuroQol questionnaire.(39)

We will collect information on surgical complications and major adverse events including infection and deep vein thrombosis, and on hospital readmissions.

Search strategy

Online databases to be searched from inception are MEDLINE, Embase, and PsycINFO on Ovid, CINAHL on EBSCOhost and the Cochrane Library. Searches of PsycINFO and CINAHL are unlikely to identify further RCTs but are routinely searched in our department. Online databases will be searched so that searches are up to date in October 2020. The search strategy for application in MEDLINE shown in Table 2 will be tailored to each database. As well as RCTs, our searches will identify systematic reviews which will be screened for RCTs as will reference lists of relevant RCTs. Citations of key articles will be tracked in Web of Science. We will identify clinical trial records in the Cochrane Library and check them for full publication. Should no further publication be identified, we will contact authors for details of progress and study results.

No language restrictions will be applied to searches or study inclusion. Translations will be made by colleagues or professional translators when required. Studies that are unobtainable through our library (including inter-library loans) and via author contact will be excluded. If studies have not reported any follow-up data or reporting is limited regarding outcomes and study conduct, including conference abstracts, we will contact authors for appropriate data. If not available, these studies will be excluded. A summary table of excluded RCTs will be produced.

Data management

Selection of studies

Records will be imported into Endnote X9 (Clarivate Analytics). An initial screen by one reviewer will exclude clearly irrelevant articles. Subsequently, abstracts and full articles will be screened independently in Covidence® by two reviewers and reasons for exclusion recorded. Discrepancies between reviewers will be resolved by consensus with involvement of an orthopaedic surgeon or methodologist. Authors will be contacted by email to confirm eligibility if necessary. Multiple reports of RCTs will be grouped together as a single study defined by a key publication with follow-up data. Notices of errata and retractions will be sought.

Data extraction

For peer review only

After piloting of forms, data will be extracted into Covidence®, Microsoft Access or Excel by one reviewer. Data to be extracted will be: country; dates of recruitment; participant characteristics including indication, age and sex; inclusion and exclusion criteria; knee replacement constructs defined by bearing mobility, constraint, fixation, bearing materials and patella resurfacing; other surgical methods including approach, alignment, computer navigation and robot assistance; rehabilitation regime; study setting to include number of surgeons and centres; sponsorship; follow-up intervals; outcome data; and information to assess risk of bias. For the outcome of revision, we will collect the number of events recorded up to a particular follow up or mean follow-up time. For continuous outcomes, we will extract means and standard deviations or, if unavailable, make estimates based on medians, interquartile ranges and ranges.(31) We will contact authors of eligible studies by email to maximise available detail concerning risk of bias, effect estimates and measures of variability for the outcomes of interest.

Depending on the number of studies identified, study characteristics will be fully extracted or checked against source material by a second reviewer. Outcome data will be extracted independently by two reviewers.

Risk of bias assessment

Risk of bias of eligible RCTs will be assessed with the revised Cochrane tool (RoB 2).(40) Protocols will be sought to identify evidence of selective outcome reporting. Assessments will be made for all RCTs by two reviewers working independently with disagreements resolved with other members of the review team. Risk of bias for individual studies will be assessed as low, some concerns, or high risk of bias. Aspects of risk of bias will be considered in both meta-analysis and narrative synthesis. Data analysis in all meta-analyses will exclude studies assessed at high risk of bias.

Data analysis

Pairwise meta-analysis

Data synthesis for the different knee replacement constructs defined by constraint, mobility, fixation, and material will start with a tabulation of study details and narrative synthesis. The unit of analysis will be the knee.

Data will be analysed in three different time periods with the cut-offs of the period time-points varied in sensitivity analysis. Initially, the periods at risk will be: “early stage failure” when the construct fails within the first three years following knee replacement; “medium stage failure” when the construct survives the early period but fails within ten years from the primary

For peer review only

surgery; and "late-stage failure" when the construct first fails ten or more years after the primary surgery.

Data analyses will start with standard meta-analysis for each pairwise comparison of knee replacement constructs. The effect measure for revision will be the hazard rate ratio. Patient-reported and surgeon-assessed outcomes are likely to be continuous outcome scores, which will be analysed using difference in mean or standardised difference in mean scores.

If the pairwise meta-analyses include ten or more RCTs we will produce funnel plots and check for asymmetry which may reflect publication bias.(41) If asymmetry is noted we will perform sensitivity analyses with exclusion of small studies, or conduct meta-regression.(31)

Network meta-analysis

We will construct a network of studies comparing different knee replacement constructs and use network meta-analysis to estimate treatment effects informed by both direct comparisons within trials and indirect comparisons across trials. Hazard ratios over the three separate time periods (early, medium, and late-stage failure) will be assumed piecewise constant but the hazard ratio of the latter two periods will be normally distributed around that of the prior period.(42) These methods will broadly follow those we have used previously.(43,44)

Network plots will be generated for each outcome to illustrate which interventions are compared directly and indirectly, and the strength of the direct and indirect evidence including the number of studies and patients available for each direct comparison. Network meta-analysis will be implemented in a Bayesian framework using OpenBUGS software (v3.2.3).(45) In our main analysis, each construct will be considered a separate intervention forming a distinct node in the network of evidence. However, this may result in a disconnected network or imprecise effect estimates. We will therefore also fit a component network meta-analysis model which assumes additivity of the components of each construct.(46) The additivity assumption will be assessed by comparing model fit between the model where each construct is distinct and the additive model.

Heterogeneity and subgroup analyses

We aim to fit both fixed and random effects models where possible, and assess heterogeneity by inspection of the between studies standard deviation and comparison of model fit between the fixed and random effects models.(31,47) However, we anticipate that there may be too few replications of individual comparisons to fit a random effects model, in which case we will implement an informative, evidence-based prior distribution for the heterogeneity variance.(48) Model fit is measured by the posterior mean residual deviance

For peer review only - http://bmjopen.bmj.com/site/about/guidelines.xhtml

(which we expect to be similar to the number of data points), and the deviance information criteria(49) (which penalises fit with a measure of model complexity), with a preference for models where these measures are smaller (where differences of at least three are considered meaningful). If data allow, we will investigate whether revision rates vary according to participant, knee replacement construct and trial characteristics (including concerns relating to risk of bias), and surgical methods including approach, alignment, computer navigation and robot assistance.

If we observe a high level of heterogeneity and cannot explain it by patient, study or surgical factors, results from the random-effects meta-analysis will be reported. If no evidence of heterogeneity is found, we will report results from the fixed effect model.

We will assess consistency between direct and indirect evidence by comparing the fit of the consistency model with the fit of an inconsistency model (the unrelated mean effects model), which relaxes the consistency assumption. Model fit statistics will be compared and the contribution to the posterior mean residual deviance for each study will be plotted for the consistency model against the inconsistency model, to identify any particular studies contributing to inconsistency.(47)

Confidence in the evidence

Our confidence in the evidence for each outcome for each intervention will be assessed using an extension of the Grading of Recommendations Assessment, Development, and Evaluation (GRADE) framework developed for network meta-analysis and implemented in the CINeMA tool.(50,51)

Systematic review 2: UKR and TKR

The systematic review and meta-analysis methods used comparing UKR with TKR in Review 2 will be similar to those used in Review 1.

Eligibility criteria

Types of studies

We will include RCTs.

Participants

Eligible patients will be as described in Review 1 but will have been assessed as eligible for both an elective UKR and an elective TKR.

Interventions and comparators

For peer review only - http://bmjopen.bmj.com/site/about/guidelines.xhtml

The knee replacement constructs will include all components used in medial, lateral and patellofemoral UKR and in TKR.

To complete the network of studies in Review 2, we will include studies comparing:

2a) different types of UKR constructs

2b) UKR with TKR constructs

2c) TKR constructs for patients eligible for UKR

Ascertaining the eligibility of trial participants for c) will be challenging due to paucity of information on patient characteristics described at trial level (e.g. number of symptomatic compartments) and time trends in clinical practice. Clinicians in our team will assess which trials may “possibly include” patients potentially eligible for UKR in a gradient up to studies which “definitely exclude” patients eligible for UKR.

Types of outcome measures

Outcome measures will be as described in Review 1.

Search strategy

The search in Review 1 includes terms for UKR and thus will identify relevant studies. Methods used will mirror those of Review 1.

Data management

Management of study information, screening and data extraction will be as described in Review 1. Data extraction will additionally include details of whether the UKR is for treatment of osteoarthritis in the medial, lateral or patellofemoral compartment.

Risk of bias assessment

Assessment of risk of bias will use methods described in Review 1.

Data analysis

For pairwise and network meta-analyses, we plan main analyses restricted to studies 2a) comparing different UKR constructs and 2b) comparing UKR and TKR constructs. Secondary analyses 2c) will also include comparisons of different TKR constructs in patients eligible for UKR.

For Review 2a), the reference construct will be the most widely used cemented mobile bearing. In Reviews 2b) and 2c) the reference construct within the network meta-analysis will be the TKR combination of cemented, cruciate retaining, fixed bearing implant components with metal on polyethylene bearing materials.

For peer review only - http://bmjopen.bmj.com/site/about/guidelines.xhtml

The methods for analysis will otherwise be as in Review 1.

Joint registry analysis

Joint registries

In our previous research(52) we have established close links between the National Joint Registry for England, Wales, Northern Ireland and the Isle of Man (NJR) and the Swedish Hip Arthroplasty Register (SHAR).(53) This was a successful collaboration and our analyses benefitted from the use of similar data collection and management in the two databases and the long follow-up of patients in Sweden. Recognising this, we have established links with the Swedish Knee Arthroplasty Register (SKAR).(54)

Data collection in the NJR commenced on April 1st, 2003 and includes 1,193,830 primary knee replacements with verifiable patient data up to 31st December 2018.(1) In 2020, up to 17 years of patient follow-up data is available for analysis.

SKAR was established in 1975 and includes patient identifier numbers which allows tracking of other healthcare use.(55) More complete data with information required for our study has been collected since 1989. Thus, in 2020, the registry includes up to 30 years of patient follow-up data.

Eligibility criteria

Patient group

Patients included in analyses will be aged 18 years or over, undergoing primary TKR or UKR for osteoarthritis, with valid data on age, sex and construct characteristics.

Interventions and comparisons

Knee replacement constructs for comparison will be characterised by bearing mobility, constraint, fixation and bearing materials. Patella resurfacing is recorded in the NJR but is infrequently used in Sweden. We will exclude patients receiving patellofemoral and bicompartmental knee replacement as they are generally used in a selected patient group.

The reference construct will be TKR with cemented, cruciate retaining, fixed bearing implant components with metal on polyethylene bearing materials.

Outcomes

Outcomes for analyses will be time to first, second and subsequent revision surgeries, and death after primary surgery.

Statistical analysis

For peer review only - http://bmjopen.bmj.com/site/about/guidelines.xhtml

We will prepare a joint statistical analysis plan for analysis of NJR and SKAR data for our research questions. The analysis plan will include: definitions of primary surgery, knee replacement constructs and techniques, follow-up time frames and age groups; statistical methods including handling of missing data; and patient characteristics for a possible subgroup of TKR patients eligible for UKR. We will estimate hazard rates of first and second revision, hazard ratios between implants, and the effect of covariates (e.g. age, gender, time since primary surgery, time since first revision) on these parameters.

The analyses will be performed using STATA software. Estimates from the SKAR population will be calibrated to the NJR population using a range of calibration models implemented in OpenBUGs software as in our previous study.(52) These calibrations will model some difference between SKAR and NJR estimates of hazard rates and hazard ratios which is either fixed, random, or independent over time periods.

Cost-effectiveness analysis

Economic decision models

RCT and registry data will be combined with cost and QALY data to inform a probabilistic lifetime decision economic model to determine the relative cost-effectiveness from an NHS and social services perspective of 1) TKR constructs and 2) TKR and UKR constructs for patients eligible for both. Results will be stratified by sex and age-group. We will limit knee replacement constructs to those available for clinical use in the NHS.

We will calculate expected costs and QALYs, discounted at 3.5% per annum using a fully probabilistic analysis, which reflects parameter uncertainty in the sampled distributions, and simulating some number of iterations. We will first use 10,000 iterations and check if it is sufficient for the mean and standard deviation of the cost and QALYs to converge; more simulations will be employed if needed. The reference construct for both models will be TKR with cemented, cruciate retaining, fixed bearing implant components with metal on polyethylene bearing materials.

The economic models will be implemented in the open source statistical software R.(56) The R software has well documented advantages of speed, flexibility, and transparency over other software such as Excel.(57) The same models will be fitted to both decision populations. Two model structures will be explored (full details are included in Supplementary material). First is a cohort Markov multistate model with health states representing time since primary surgery, post first revision, post second or higher revision, and death.(58) States for time since primary will correspond to <3 years, ≥3 years but <10 years, and ≥10 years post primary. The post 1st revision states will correspond to earl (<3

For peer review only - http://bmjopen.bmj.com/site/about/guidelines.xhtml

years), middle (≥3 years but <10 years), and late (≥10 years) revision. Probabilities of 1st revision will depend on the time since primary while probabilities of 2nd revision will depend on whether the 1st revision was early, middle, or late.

The second model structure we will use is an individual level continuous time semi-Markov model.(59) We aim to employ the 'hesim' extension to R to implement this model.(60) States will correspond to no revision, post 1st revision, post 2nd revision, and death. Transition rates of 1st revision will depend on time since primary surgery and patient characteristics (e.g. age and gender). Rates of 2nd revision will depend on time from primary to 1st revision and time since 1st revision. This is a refinement of the cohort Markov multistate model and results are expected to be similar. Our base case will be the individual level continuous time semi-Markov model as it places fewer restrictions on timings of events.

We will conduct a literature search to identify the best data source to derive quality adjusted life years (QALYs) stratified by age and sex for use in the models. A potential source is the UK Patient Reported Outcome Measures (PROMs) dataset for patients with knee replacement.(61) The PROMs dataset includes EQ-5D-3L data at six months post-surgery for patients over 40 years old.

Implant prices in GB pounds will be estimated from a national database of NHS trusts providing implant procurement prices to the NJR: the NJR INFORM Implant price-benchmarking database.(62) Primary and revision surgeries, and follow-up healthcare costs will be obtained from national tariffs.(63) We will search the literature for published estimates of primary and secondary care follow-up costs from cost-effectiveness analyses in RCTs of knee replacement.

Cost-effectiveness will be estimated using the mean incremental net monetary benefit statistic (INMB) for each knee replacement construct compared to the reference construct, a willingness-to-pay threshold of £20,000 per QALY.(64) The construct with the highest INMB is the most cost-effective for each patient subgroup. With cost-effectiveness acceptability curves we will show how the probability of knee replacement constructs being most cost-effective varies as willingness-to-pay thresholds change.

In sensitivity analyses we will assess the robustness of the results to changes in key parameters and assumptions. These will involve variation of: time periods over which event hazards are assumed constant; estimations of transition probabilities (network meta-analysis only, registries only, combination of both); model structure to include different health states; costs of constructs; time in theatre to perform different surgeries; revision TKR costs; and other follow-up costs.

For peer review only - http://bmjopen.bmj.com/site/about/guidelines.xhtml

Ethics and dissemination

The systematic reviews will not require ethical approval as we are undertaking secondary analyses of published data. For the analyses of joint registry data, we will design a common analysis plan and provide representatives of the NJR and SKAR with information required for submission to their respective ethics committees. The project has been assessed with the NHS REC committee and does not require its ethical review.

We expect the results from our study to provide direct patient benefit through better information available for clinical surgical teams performing TKRs, which will influence the commissioning of knee replacement constructs used at NHS trusts in the UK. We will publish our effectiveness and cost-effectiveness findings in two separate peer-reviewed journal articles in high impact open access journals. The findings of our studies will also be disseminated to the NHS, NJR, clinicians, societies, charities, patients and the general public via presentations, reports and websites.

Discussion

Surgeons and patients are faced with many construct and technique options for use in knee replacement. Patients in our PPI group told us that they were not informed about surgical choices when discussing surgery with their consultant. In a stakeholders meeting, knee surgeons advised us that they chose knee replacement constructs based on their preferences, surgical skills, availability of implants from manufacturers and available research evidence. There is a need for a comprehensive synthesis of evidence to inform this choice.

Given the large number of knee replacements performed annually and stringent NHS budgets, it is important to determine which knee replacement constructs provide the best outcomes for patients at lowest cost to the NHS. Ideally, all constructs would be compared in an RCT with long-term follow-up and enough statistical power to estimate differences in revision rates between constructs. However, it would be difficult to compare large numbers of constructs in an RCT and fund long-term follow-up.

We suggest that a more practical and efficient approach is to use all available evidence in published head-to-head RCTs to obtain treatment effect estimates for knee replacement constructs. We will complement these with analyses of large longitudinal national registries of surgical and patient data with long-term follow-up. Both estimates will be used to produce economic decision models which will rank and estimate the cost-effectiveness of knee replacement constructs.

For peer review only - http://bmjopen.bmj.com/site/about/guidelines.xhtml

Author statement

Elsa M R Marques: chief investigator; conduct network meta-analysis; perform Markov economic model analyses; write articles and lead dissemination.

Jane Dennis: lead systematic reviewer

Andrew D Beswick: secondary systematic reviewer; aid in writing of articles and dissemination.

Julian PT Higgins: provide methodological expertise in evidence synthesis; supervise systematic review and network meta-analysis.

Howard Thom: support for network meta-analysis, analysis of joint registries and economic models.

Nicky J Welton: methodological support for network meta-analysis and economics models; support dissemination of results.

Amanda Burston: lead PPI activities

Linda P Hunt: aid in producing analysis plans for NJR and SKAR; analysis of NJR data.

Michael R Whitehouse: clinical support; aid in writing of articles and dissemination.

Ashley W Blom: clinical advice; liaise with SKAR; support dissemination of results.

Data statement

All relevant data identified in the systematic review will be included in the published final report and supplementary material.

Funding source

This study is funded by the National Institute for Health Research (NIHR) Research for Patient Benefit programme (project reference PB-PG-1013-32010). The views expressed are those of the authors and not necessarily those of the NIHR or the Department of Health and Social Care.

The funder had no role in the design of the study and collection, analysis, and interpretation of data and in writing the manuscript. The researchers were independent from funders and all authors had full access to all of the data in the study and can take responsibility for the integrity of the data and the accuracy of the data analysis. Funding for Review 2 is applied for.

Competing interests

For peer review only

All authors declare no conflicts of interest.

For peer review only

References

1. National Joint Registry for England, Wales, Northern Ireland and the Isle of Man: 16th Annual report. Hemel Hempstead: NJR Centre 2019.

2. Scottish Arthroplasty Project. Annual report 2019. Edinburgh: NHS National Services Scotland; 2019.

3. Swedish Knee Arthroplasty Register: Annual report 2019. Lund: Skåne University Hospital; 2019.

4. Evans JT, Walker RW, Evans JP, Blom AW, Sayers A, Whitehouse MR. How long does a knee replacement last? A systematic review and meta-analysis of case series and national registry reports with more than 15 years of follow-up. Lancet. 2019;393(10172):655-63.

5. Willis-Owen CA, Brust K, Alsop H, Miraldo M, Cobb JP. Unicondylar knee arthroplasty in the UK National Health Service: An analysis of candidacy, outcome and cost efficacy. Knee. 2009;16(6):473-8.

6. Wynn Jones H, Chan W, Harrison T, Smith TO, Masonda P, Walton NP. Revision of medial Oxford unicompartmental knee replacement to a total knee replacement: Similar to a primary? Knee. 2012;19(4):339-43.

7. Ma J-x, He W-w, Kuang M-j, Sun L, Lu B, Wang Y, et al. Efficacy of bicompartmental knee arthroplasty (BKA) for bicompartmental knee osteoarthritis: A meta analysis. Int J Surg. 2017;46:53-60.

8. Arirachakaran A, Sangkaew C, Kongtharvonskul J. Patellofemoral resurfacing and patellar denervation in primary total knee arthroplasty. Knee Surg Sports Traumatol Arthrosc. 2015;23(6):1770-81.

9. Liu CY, Li CD, Wang L, Ren S, Yu FB, Li JG, et al. Function scores of different surgeries in the treatment of knee osteoarthritis: A PRISMA-compliant systematic review and network-meta analysis. Medicine. 2018;97(21):e10828.

10. Singh JA, Tugwell P, Zanoli G, Wells GA. Total joint replacement surgery for knee osteoarthritis and other non-traumatic diseases: A network meta-analysis. Cochrane Database Syst Rev. 2015;2015 (6)(CD011765).

11. Singh JA, Tugwell P, Zanoli G, Wells GA. Total joint replacement surgery for knee osteoarthritis and other non-traumatic diseases: a network meta-analysis. Cochrane Database Syst Rev. 2019(9).

12. Meijer KA, Dasa V. Is resurfacing the patella cheaper? An economic analysis of evidence based medicine on patellar resurfacing. Knee. 2015;22(2):136-41.

13. Daigle ME, Weinstein AM, Katz JN, Losina E. The cost-effectiveness of total joint arthroplasty: a systematic review of published literature. Best Pract Res Clin Rheumatol. 2012;26(5):649-58.

For peer review only - http://bmjopen.bmj.com/site/about/guidelines.xhtml

14. Burn E, Liddle AD, Hamilton TW, Pai S, Pandit HG, Murray DW, et al. Choosing between unicompartmental and total knee replacement: What can economic evaluations tell us? A systematic review. Pharmacoeconom Open. 2017;1(4):241-53.
15. Chawla H, Ghomrawi HM, van der List JP, Eggman AA, Zuiderbaan HA, Pearle AD. Establishing age-specific cost-effective annual revision rates for unicompartmental knee arthroplasty: A meta-analysis. J Arthroplasty. 2017;32(1):326-35.
16. Smith WB, Steinberg J, Scholtes S, McNamara IR. Medial compartment knee osteoarthritis: age-stratified cost-effectiveness of total knee arthroplasty, unicompartmental knee arthroplasty, and high tibial osteotomy. Knee Surg Sports Traumatol Arthrosc. 2017;25(3):924-33.
17. Kamaruzaman H, Kinghorn P, Oppong R. Cost-effectiveness of surgical interventions for the management of osteoarthritis: a systematic review of the literature. BMC Musculoskelet Disord. 2017;18(1):183.
18. Fennema P, Heyse TJ, Uyl-de Groot CA. Cost-effectiveness and clinical implications of advanced bearings in total knee arthroplasty: a long-term modeling analysis. Int J Technol Assess Health Care. 2014;30(2):218-25.
19. Gioe T, Sharma A, Tatman P, Mehle S. Do "premium" joint implants add value? Analysis of high cost joint implants in a community registry (Provisional abstract). Clin Orthop Relat Res. 2011;469(1):48-54.
20. Partridge TCJ, Baker PN, Jameson SS, Mason J, Reed MR, Deehan DJ. Conventional versus highly cross-linked polyethylene in primary total knee replacement: A comparison of revision rates using data from the National Joint Registry for England, Wales, and Northern Ireland J Bone Joint Surg Am. 2019;102(2):119-27.
21. Gioe TJ, Sinner P, Mehle S, Ma W, Killeen KK. Excellent survival of all-polyethylene tibial components in a community joint registry. Clin Orthop Relat Res. 2007;464:88-92.
22. Weeks CA, Marsh JD, MacDonald SJ, Graves S, Vasarhelyi EM. Patellar resurfacing in total knee arthroplasty: A cost-effectiveness analysis. J Arthroplasty. 2018;33(11):3412-5.
23. Manoli A, Markel JF, Pizzimenti NM, Markel DC. Early results of a modern uncemented total knee arthroplasty system. Orthopedics. 2019;42(6):355-60.
24. Chawla H, Nwachukwu BU, van der List JP, Eggman AA, Pearle AD, Ghomrawi HM. Cost effectiveness of patellofemoral versus total knee arthroplasty in younger patients. Bone Joint J. 2017;99-B(8):1028-36.
25. Peersman G, Jak W, Vandenlangenbergh T, Jans C, Cartier P, Fennema P. Cost-effectiveness of unicondylar versus total knee arthroplasty: a Markov model analysis. Knee. 2014;21 Suppl 1:S37-42.
26. Burn E, Liddle AD, Hamilton TW, Judge A, Pandit HG, Murray DW, et al. Cost-effectiveness of unicompartmental compared with total knee replacement: a population-

For peer review only - http://bmjopen.bmj.com/site/about/guidelines.xhtml

based study using data from the National Joint Registry for England and Wales. BMJ Open. 2018;8(4):e020977.
27. Kolovos S, Strauss VY, Prats-Uribe A, Prieto-Alhambra D, Pinedo-Villanueva R. Hospital costs and outcomes of unicompartemental compared to total knee replacement for patients with multiple comorbidities: A population-based study. Value Health. 2019;22 (Supplement 3):S921.
28. Pennington M, Grieve R, Black N, van der Meulen JH. cost-effectiveness of five commonly used prosthesis brands for total knee replacement in the UK: A study using the NJR dataset. PLoS One. 2016;11(3):e0150074.
29. Moher D, Shamseer L, Clarke M, Ghersi D, Liberati A, Petticrew M, et al. Preferred reporting items for systematic review and meta-analysis protocols (PRISMA-P) 2015 statement. Syst Rev. 2015;4:1.
30. Richardson WS, Wilson MC, Nishikawa J, Hayward RS. The well-built clinical question: a key to evidence-based decisions. ACP J Club. 1995;123(3):A12-3.
31. Higgins JPT, Thomas J, Chandler J, Cumpston M, Li T, Page MJ, et al. Cochrane Handbook for Systematic Reviews of Interventions. 2nd Edition. Chichester (UK): John Wiley & Sons; 2019.
32. Moher D, Liberati A, Tetzlaff J, Altman DG. Preferred reporting items for systematic reviews and meta-analyses: the PRISMA statement. BMJ. 2009;339:b2535.
33. Hutton B, Salanti G, Caldwell DM, Chaimani A, Schmid CH, Cameron C, et al. The PRISMA extension statement for reporting of systematic reviews incorporating network meta-analyses of health care interventions: Checklist and explanations. PRISMA extension for network meta-analysis. Ann Intern Med. 2015;162(11):777-84.
34. Guyot P, Ades AE, Ouwens MJ, Welton NJ. Enhanced secondary analysis of survival data: reconstructing the data from published Kaplan-Meier survival curves. BMC Med Res Methodol. 2012;12:9.
35. Murray D, Fitzpatrick R, Rogers K, Pandit H, Beard D, Carr A, et al. The use of the Oxford hip and knee scores. J Bone Joint Surg Br. 2007;89-B(8):1010-4.
36. Bellamy N, Buchanan WW, Goldsmith C, Campbell J, Stitt L. Validation study of WOMAC: A health status instrument for measuring clinically important patient relevant outcomes to antirheumatic drug therapy in patients with osteoarthritis of the hip or knee. J Rheumatol. 1989;15:1833-40.
37. Insall JN, Dorr LD, Scott RD, Scott WN. Rationale of the Knee Society clinical rating system. Clin Orthop Relat Res. 1989(248):13-4.
38. Insall JN, Ranawat CS, Aglietti P, Shine J. A comparison of four models of total knee-replacement prostheses. J Bone Joint Surg Am. 1976;58(6):754-65.
39. Brooks R. EuroQol: the current state of play. Health Policy. 1996;37(1):53-72.

For peer review only - http://bmjopen.bmj.com/site/about/guidelines.xhtml

40. Sterne JAC, Savović J, Page MJ, Elbers RG, Blencowe NS, Boutron I, et al. RoB 2: a revised tool for assessing risk of bias in randomised trials BMJ 2019;366:l4898.
41. Sterne JA, Sutton AJ, Ioannidis JP, Terrin N, Jones DR, Lau J, et al. Recommendations for examining and interpreting funnel plot asymmetry in meta-analyses of randomised controlled trials. BMJ. 2011;343:d4002.
42. Lu G, Ades AE, Sutton AJ, Cooper NJ, Briggs AH, Caldwell DM. Meta-analysis of mixed treatment comparisons at multiple follow-up times. Stat Med. 2007;26(20):3681-99.
43. Marques EMR, Humphriss R, Welton NJ, Higgins JPT, Hollingworth W, Lopez-Lopez JA, et al. The choice between hip prosthetic bearing surfaces in total hip replacement: a protocol for a systematic review and network meta-analysis. Syst Rev. 2016;5(1):19.
44. Lopez-Lopez JA, Humphriss RL, Beswick AD, Thom HHZ, Hunt LP, Burston A, et al. Choice of implant combinations in total hip replacement: systematic review and network meta-analysis. BMJ. 2017;359:j4651.
45. Lunn D, Spiegelhalter D, Thomas A, Best N. The BUGS project: Evolution, critique and future directions. Stat Med. 2009;28(25):3049-67.
46. Welton NJ, Caldwell DM, Adamopoulos E, Vedhara K. Mixed treatment comparison meta-analysis of complex interventions: psychological interventions in coronary heart disease. Am J Epidemiol. 2009;169(9):1158-65.
47. Dias S, Ades AE, Welton NJ, Jansen JP, Sutton AJ. Network meta-analysis for comparative effectiveness research. Hoboken NJ: Wiley; 2018.
48. Turner RM, Davey J, Clarke MJ, Thompson SG, Higgins JP. Predicting the extent of heterogeneity in meta-analysis, using empirical data from the Cochrane Database of Systematic Reviews. Int J Epidemiol. 2012;41(3):818-27.
49. Spiegelhalter DJ, Best NG, Carlin BP, Van Der Linde A. Bayesian measures of model complexity and fit. J R Stat Soc Series B Stat Methodol. 2002;64(4):583-639.
50. Nikolakopoulou A, Higgins JPT, Papakonstantinou T, Chaimani A, Del Giovane C, Egger M, et al. CINeMA: An approach for assessing confidence in the results of a network meta-analysis. PLOS Medicine. 2020;17(4):e1003082.
51. Papakonstantinou T, Nikolakopoulou A, Higgins JPT, Egger M, Salanti G. CINeMA: Software for semiautomated assessment of the confidence in the results of network meta-analysis. Campbell Syst Rev. 2020;16(1):e1080.
52. Fawsitt CG, Thom HHZ, Hunt LP, Nemes S, Blom AW, Welton NJ, et al. Choice of prosthetic implant combinations in total hip replacement: Cost-effectiveness analysis using UK and Swedish hip joint registries data. Value Health. 2018;22:303-12.
53. Malchau H, Herberts P, Eisler T, Garellick G, Soderman P. The Swedish Total Hip Replacement Register. J Bone Joint Surg Am. 2002;84-A Suppl 2:2-20.

For peer review only - http://bmjopen.bmj.com/site/about/guidelines.xhtml

54. Knutson K, Lewold S, Robertsson O, Lidgren L. The Swedish knee arthroplasty register: A nation-wide study of 30,003 knees 1976-1992. Acta Orthop Scand. 1994;65(4):375-86.
55. Robertsson O, Ranstam J, Sundberg M, W-Dahl A, Lidgren L. The Swedish Knee Arthroplasty Register: a review. Bone Joint Res. 2014;3(7):217-22.
56. R Core Team. R: A language and environment for statistical computing. Vienna: Foundation for Statistical Computing; 2015.
57. Incerti D, Thom H, Baio G, Jansen JP. R you still using Excel? The advantages of modern software tools for health technology assessment. Value Health. 2019;22(5):575-9.
58. Siebert U, Alagoz O, Bayoumi AM, Jahn B, Owens DK, Cohen DJ, et al. State-transition modeling: a report of the ISPOR-SMDM Modeling Good Research Practices Task Force--3. Value Health. 2012;15(6):812-20.
59. Williams C, Lewsey JD, Briggs AH, Mackay DF. Cost-effectiveness analysis in R using a multi-state modeling survival analysis framework: A tutorial. Med Decis Making. 2017;37(4):340-52.
60. Incerti D, Jansen JP, R Core Team. Health-economic simulation modeling and decision analysis 2020 [Available from: https://cran.r-project.org/web/packages/hesim/hesim.pdf.
61. Finalised Patient Reported Outcome Measures (PROMs) in England for Hip and Knee Replacement procedures (April 2017 to March 2018) Leeds: NHS Digital; 2019 [Available from: https://digital.nhs.uk/data-and-information/publications/statistical/patient-reported-outcome-measures-proms/hip-and-knee-replacement-procedures---april-2017-to-march-2018.
62. Highlights: Economic model & price benchmarking. Hemel Hempstead: National Joint Registry for England, Wales, Northern Ireland and the Isle of Man; 2016.
63. NHS. NHS Reference Costs 2014 to 2015. London: Department of Health; 2015.
64. Guide to the methods of technology appraisal 2013. Process and methods. London: National Institute for Health and Care Excellence; 2013.

For peer review only - http://bmjopen.bmj.com/site/about/guidelines.xhtml

Table 1. Knee construct options

**Total knee replacement**					
Bearing mobility	Fixed bearing	Mobile bearing			
Constraint	Cruciate retaining	Posterior stabilised	Bi-cruciate stabilised	Constrained condylar	Hinged
Fixation	Cemented	Uncemented	Hybrid		
Bearing materials	Metal bearing on conventional or highly cross-linked polyethylene tray	Metal femoral component on all-polyethylene tibial component (monobloc)	Other materials (ceramic bearings, ceramicized metals)	Specialised or customised implants	
Patella resurfacing	Patella resurfacing	No patella resurfacing			
**Unicompartmental knee replacement (medial or lateral)**					
Bearing mobility	Fixed bearings	Mobile bearing			
Constraint	Cruciate retaining				
Fixation	Cemented	Uncemented	Hybrid		
Bearing materials	Metal on polyethylene	Metal femoral component (monobloc)			

For peer review only

Table 2. Search strategy as applied in MEDLINE

1	controlled clinical trial.pt.
2	randomized controlled trial.pt.
3	clinical trials as topic/
4	(randomi#ed or randomi#ation or randomi#ing).ti,ab,kf.
5	(RCT or "at random" or (random* adj3 (administ* or allocat* or assign* or class* or cluster or crossover or cross-over or control* or determine* or divide* or division or distribut* or expose* or fashion or number* or place* or pragmatic or quasi or recruit* or split or subsitut* or treat*))).ti,ab,kf.
6	placebo.ab,ti,kf.
7	trial.ti.
8	(control* adj3 group*).ab.
9	(control* and (trial or study or group*) and (waitlist* or wait* list* or ((treatment or care) adj2 usual))).ti,ab,kf.
10	((single or double or triple or treble) adj2 (blind* or mask* or dummy)).ti,ab,kf.
11	double-blind method/ or random allocation/ or single-blind method/
12	or/1-11
13	(systematic or structured or evidence or trials or studies).ti. and ((review or overview or look or examination or update* or summary).ti. or review.pt.)
14	(0266-4623 or 1469-493X or 1366-5278 or 1530-440X or 2046-4053).is.
15	meta-analysis.pt. or (meta-analys* or meta analys* or metaanalys* or meta synth* or meta-synth* or metasynth*).ti,ab,kf,hw.
16	((systematic or meta) adj2 (analys* or review)).ti,kf. or ((systematic* or quantitativ* or methodologic*) adj5 (review* or overview*)).ti,ab,kf,sh. or (quantitativ$ adj5 synthesis$).ti,ab,kf,hw.
17	(integrative research review* or research integration).tw. or scoping review?.ti,kf. or (review.ti,kf,pt. and (trials as topic or studies as topic).hw.) or (evidence adj3 review*).ti,ab,kf.
18	review.pt. and ((medline or medlars or embase or pubmed or scisearch or psychinfo or psycinfo or psychlit or psyclit or cinahl or electronic database* or bibliographic database* or computeri#ed database* or online database* or pooling or pooled or mantel haenszel or peto or dersimonian or der simonian or fixed effect or ((hand adj2 search*) or (manual* adj2 search*))).tw,hw. or (retraction of publication or retracted publication).pt.)
19	or/13-18
20	Arthroplasty, Replacement, Knee/
21	Knee Prosthesis/
22	(arthoplast$ adj3 knee$).mp. [mp=title, abstract, original title, name of substance word, subject heading word, floating sub-heading word, keyword heading word, organism supplementary concept word, protocol supplementary concept word, rare disease supplementary concept word, unique identifier, synonyms]
23	(knee$ adj3 replac$).mp. [mp=title, abstract, original title, name of substance word, subject heading word, floating sub-heading word, keyword heading word, organism supplementary concept word, protocol supplementary concept word, rare disease supplementary concept word, unique identifier, synonyms]
24	(knee$ adj3 implant$).mp. [mp=title, abstract, original title, name of substance word, subject heading word, floating sub-heading word, keyword heading word, organism supplementary concept word, protocol supplementary concept word, rare disease supplementary concept word, unique identifier, synonyms]
25	(knee$ adj3 prosthe$).mp. [mp=title, abstract, original title, name of substance word, subject heading word, floating sub-heading word, keyword heading word, organism supplementary concept word, protocol supplementary concept word, rare disease supplementary concept word, unique identifier, synonyms]
26	(knee$ adj3 endoprosthe$).mp. [mp=title, abstract, original title, name of substance word, subject heading word, floating sub-heading word, keyword heading word, organism supplementary concept word, protocol supplementary concept word, rare disease supplementary concept word, unique identifier, synonyms]
27	unicondylar.mp.
28	unicompartmental.mp.
29	or/20-28
30	12 or 19
31	29 and 30

For peer review only

For peer review only - http://bmjopen.bmj.com/site/about/guidelines.xhtml

Supplementary material. Economic decision models

Model structure

Potential model structures were developed by the research team with the help of surgeons, patients, and searches for previous economic models. We will start by applying a Markov decision model with tunnel states to reflect the three different time periods at risk, as depicted in Figure 1.

Figure 1. A Markov model using tunnel states to model outcomes after total knee replacement (`A').*

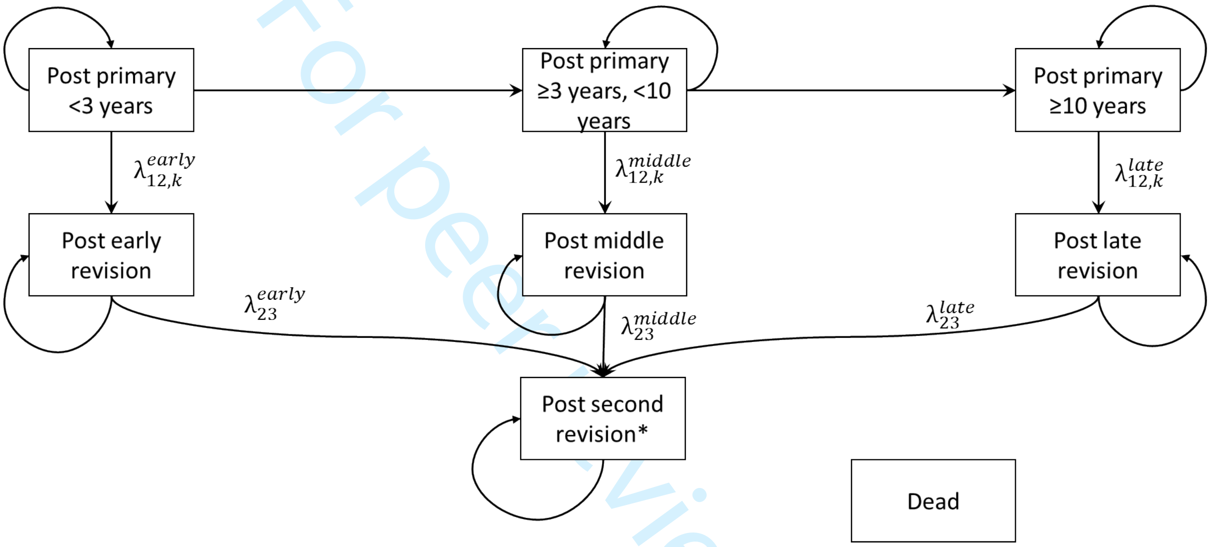

*Indices of transition rates λ_{ij} correspond to sets of states i and j: "Post TKR" ($i = 1$), Post revision ($i = 2$), and post second revision ($i = 3$).

States are divided into three groups corresponding to post primary (set 1), post revision (set 2), and post second revision (set 3) surgery. This model splits revisions into three time periods: up to 3 years post primary surgery ("early"), between 3- and 10-years post-primary ("middle"), and 10 or more years post primary surgery ("late"). This division applies to the post primary and post revision sets of states.

The three post primary states have three log hazard rates of revision $\lambda_{12,k}^{early}$, $\lambda_{12,k}^{middle}$, and $\lambda_{12,k}^{late}$, for implant k, such as:

Equation 1. $\lambda_{12,k}^{period} = \alpha_{12}^{period} + d_{12,k}^{period}$

In Equation 1, period is "early", "middle", or "late" first revision.

α_{12}^{period} is the log hazard of 1st revision for the baseline implant and informed by NJR or SKAR data, which is constant over time within each 'period'. $d_{12,k}^{period}$ is the log hazard ratio of 1st revision for implant k relative to the baseline and it is informed by the results of the NMA,

For peer review only - http://bmjopen.bmj.com/site/about/guidelines.xhtml

combined with NJR or SKAR data. If NMA estimates are too sparse or uncertain, will use observational estimates from registries only.

These time-independent log rates are converted to annual probabilities for the 1-year cycle length using the exponential distribution

Equation 2. $P_{12,k}^{period} = 1 - \exp\left(-\exp\left(\lambda_{12,k}^{period}\right)\right)$

The rates of second revision are assumed to depend on whether the first revision occurred in the "early", "middle", or "late" first revision period, with constant log hazards λ_{23}^{early}, λ_{23}^{middle}, and λ_{23}^{late}. These are again converted to probabilities using an exponential distribution

Equation 3. $P_{23}^{period} = 1 - \exp\left(-\exp\left(\lambda_{23}^{period}\right)\right)$

The log rates of second revision will be estimated using NJR and SKAR data and will be assumed to be the same for all implants (hence no index k); note that they are different to the log rates of first revision.

The annual probability of death $P_M(u)$ will be assumed independent of current state or implant. It will depend on current time u as it will increase with age as in the Office of National Statisticians' life tables. Dependence of all log hazards of 1st and 2nd revision on age and gender will be managed through stratification into male/female and age bands (e.g. <55, 55-64, 65-74, 75+ years old).

We will also explore using an individual level continuous time semi-Markov multistate model, which simulates individual patient paths in continuous time.

A continuous model allows the hazard rate and hazard rate ratios of first revision to vary continuously, giving a log transition rate of 1st revision function $\lambda_{12,k}(u,x_i)$ that depends on current time u and, possibly, on patient characteristics x_i. The constant hazards over the early, middle, and late periods in the cohort Markov model are an approximation to this function. The log rate $\lambda_{12,k}(u,x_i)$ is generated using baseline implant hazards $\alpha_{12}(u,x_i)$ (i.e. $k = 1$) from NJR/SKAR and hazard ratios $d_{12,k}(u,x_i)$ from NJR/SKAR and the NMA for other implants k:

Equation 4. Individual patient semi-Markov : $\lambda_{12,k}(u,x_i) = \alpha_{12}(u,x_i) + d_{12,k}(u,x_i)$

Unlike the cohort Markov model the log rates of revision on the baseline implant $\alpha_{12}(u,x_i)$ and log hazard ratios $d_{12,k}(u,x_i)$ for implant k are now functions (rather than three constants).

The hazard of second revision $\lambda_{23}(u,t_{12,i})$ will depend on the time taken by individual patient i to experience first revision $t_{12,i}$ (i.e. time to transition to from 1st to 2nd state). Mortality is

For peer review only - http://bmjopen.bmj.com/site/about/guidelines.xhtml

modelled as a log hazard of death $\lambda_M(u,x_i)$ which is assumed independent of current state or implant but may depend on patient characteristics x_i (e.g. age and gender)

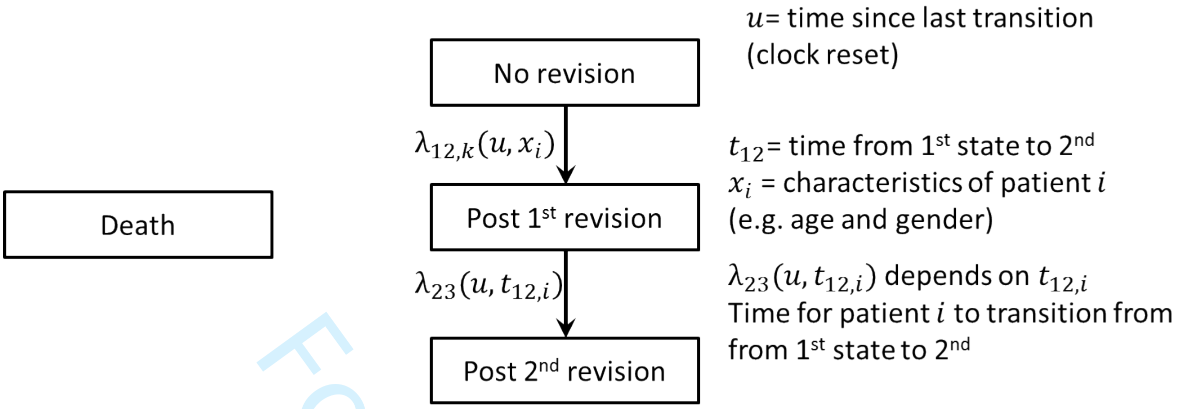

For peer review only

For peer review only - http://bmjopen.bmj.com/site/about/guidelines.xhtml

Supplementary material 1. PRISMA-P (Preferred Reporting Items for Systematic review and Meta-Analysis Protocols) 2015 checklist: recommended items to address in a systematic review protocol*

Section and topic	Item No	Checklist item	
**ADMINISTRATIVE INFORMATION**			
Title:			
Identification	1a	Identify the report as a protocol of a systematic review	Title
Update	1b	If the protocol is for an update of a previous systematic review, identify as such	N/A
Registration	2	If registered, provide the name of the registry (such as PROSPERO) and registration number	Provided
Authors:			
Contact	3a	Provide name, institutional affiliation, e-mail address of all protocol authors; provide physical mailing address of corresponding author	Provided
Contributions	3b	Describe contributions of protocol authors and identify the guarantor of the review	Provided
Amendments	4	If the protocol represents an amendment of a previously completed or published protocol, identify as such and list changes; otherwise, state plan for documenting important protocol amendments	N/A
Support:			
Sources	5a	Indicate sources of financial or other support for the review	NIHR funding described
Sponsor	5b	Provide name for the review funder and/or sponsor	The study is registered with the funder (the NIHR), the host (the NHS North Bristol Trust) and the sponsor (University of Bristol) and has obtained all approvals to start
Role of sponsor or funder	5c	Describe roles of funder(s), sponsor(s), and/or institution(s), if any, in developing the protocol	Described in “Funding source”
**INTRODUCTION**			
Rationale	6	Describe the rationale for the review in the context of what is already known	Introduction
Objectives	7	Provide an explicit statement of the question(s) the review will address with reference to participants, interventions, comparators, and outcomes (PICO)	Introduction

For peer review only - http://bmjopen.bmj.com/site/about/guidelines.xhtml

For peer review only

**METHODS**			
Eligibility criteria	8	Specify the study characteristics (such as PICO, study design, setting, time frame) and report characteristics (such as years considered, language, publication status) to be used as criteria for eligibility for the review	Methods: Eligibility criteria
Information sources	9	Describe all intended information sources (such as electronic databases, contact with study authors, trial registers or other grey literature sources) with planned dates of coverage	Methods: Eligibility criteria
Search strategy	10	Present draft of search strategy to be used for at least one electronic database, including planned limits, such that it could be repeated	Table 2
Study records:			
Data management	11a	Describe the mechanism(s) that will be used to manage records and data throughout the review	Methods: data management
Selection process	11b	State the process that will be used for selecting studies (such as two independent reviewers) through each phase of the review (that is, screening, eligibility and inclusion in meta-analysis)	Methods: data management
Data collection process	11c	Describe planned method of extracting data from reports (such as piloting forms, done independently, in duplicate), any processes for obtaining and confirming data from investigators	Methods: data management
Data items	12	List and define all variables for which data will be sought (such as PICO items, funding sources), any pre-planned data assumptions and simplifications	Methods: data management
Outcomes and prioritization	13	List and define all outcomes for which data will be sought, including prioritization of main and additional outcomes, with rationale	Introduction: objectives<br>Methods: types of outcome measures
Risk of bias in individual studies	14	Describe anticipated methods for assessing risk of bias of individual studies, including whether this will be done at the outcome or study level, or both; state how this information will be used in data synthesis	Methods: risk of bias assessment
Data synthesis	15a	Describe criteria under which study data will be quantitatively synthesised	Methods; pairwise meta-analysis<br>Methods: network meta-analysis
	15b	If data are appropriate for quantitative synthesis, describe planned summary measures, methods of handling data and methods of combining data from studies, including any planned exploration of consistency (such as I^2, Kendall's τ)	Methods; pairwise meta-analysis<br>Methods: network meta-analysis
	15c	Describe any proposed additional analyses (such as sensitivity or subgroup analyses, meta-regression)	Methods: Heterogeneity and subgroup analyses
	15d	If quantitative synthesis is not appropriate, describe the type of summary planned	Methods: pairwise meta-analysis

For peer review only - http://bmjopen.bmj.com/site/about/guidelines.xhtml

Meta-bias(es)	16	Specify any planned assessment of meta-bias(es) (such as publication bias across studies, selective reporting within studies)	Methods: risk of bias assessment<br>Methods: pairwise meta-analysis
Confidence in cumulative evidence	17	Describe how the strength of the body of evidence will be assessed (such as GRADE)	Methods: quality of evidence

*** It is strongly recommended that this checklist be read in conjunction with the PRISMA-P Explanation and Elaboration (cite when available) for important clarification on the items. Amendments to a review protocol should be tracked and dated. The copyright for PRISMA-P (including checklist) is held by the PRISMA-P Group and is distributed under a Creative Commons Attribution Licence 4.0.**

From: Shamseer L, Moher D, Clarke M, Ghersi D, Liberati A, Petticrew M, Shekelle P, Stewart L, PRISMA-P Group. Preferred reporting items for systematic review and meta-analysis protocols (PRISMA-P) 2015: elaboration and explanation. BMJ. 2015 Jan 2;349(jan02 1):g7647.

For peer review only

For peer review only - http://bmjopen.bmj.com/site/about/guidelines.xhtml

BMJ Open

The choice between implants in knee replacement: Protocol for a Bayesian network meta-analysis, analysis of joint registries and economic decision model to determine the effectiveness and cost-effectiveness of knee implants for NHS patients. The KNee Implant Prostheses Study (KNIPS)

Journal:	*BMJ Open*
Manuscript ID	bmjopen-2020-040205.R1
Article Type:	Protocol
Date Submitted by the Author:	16-Nov-2020
Complete List of Authors:	Marques, Elsa; Bristol Medical School, Musculoskeletal Research Unit<br>Dennis, Jane; Bristol Medical School, Musculoskeletal Research Unit<br>Beswick, Andrew; Bristol Medical School, Musculoskeletal Research Unit<br>Higgins, Julian; Bristol Medical School, Population Health Sciences<br>Thom, H; Bristol Medical School, Population Health Sciences<br>Welton, Nicky; Bristol Medical School, Population Health Sciences<br>Burston, Amanda; Bristol Medical School, Musculoskeletal Research Unit<br>Hunt, Linda; Bristol Medical School, Musculoskeletal Research Unit<br>Whitehouse, Michael; Bristol Medical School, Musculoskeletal Research Unit<br>Blom, AW; Bristol Medical School, Musculoskeletal Research Unit
**Primary Subject Heading**:	Health economics
Secondary Subject Heading:	Evidence based practice, Surgery, Rheumatology
Keywords:	Knee < ORTHOPAEDIC & TRAUMA SURGERY, Adult orthopaedics < ORTHOPAEDIC & TRAUMA SURGERY, STATISTICS & RESEARCH METHODS, HEALTH ECONOMICS

SCHOLARONE™
Manuscripts

I, the Submitting Author has the right to grant and does grant on behalf of all authors of the Work (as defined in the below author licence), an exclusive licence and/or a non-exclusive licence for contributions from authors who are: i) UK Crown employees; ii) where BMJ has agreed a CC-BY licence shall apply, and/or iii) in accordance with the terms applicable for US Federal Government officers or employees acting as part of their official duties; on a worldwide, perpetual, irrevocable, royalty-free basis to BMJ Publishing Group Ltd ("BMJ") its licensees and where the relevant Journal is co-owned by BMJ to the co-owners of the Journal, to publish the Work in this journal and any other BMJ products and to exploit all rights, as set out in our licence.

The Submitting Author accepts and understands that any supply made under these terms is made by BMJ to the Submitting Author unless you are acting as an employee on behalf of your employer or a postgraduate student of an affiliated institution which is paying any applicable article publishing charge ("APC") for Open Access articles. Where the Submitting Author wishes to make the Work available on an Open Access basis (and intends to pay the relevant APC), the terms of reuse of such Open Access shall be governed by a Creative Commons licence – details of these licences and which Creative Commons licence will apply to this Work are set out in our licence referred to above.

Other than as permitted in any relevant BMJ Author's Self Archiving Policies, I confirm this Work has not been accepted for publication elsewhere, is not being considered for publication elsewhere and does not duplicate material already published. I confirm all authors consent to publication of this Work and authorise the granting of this licence.

For peer review only

The choice between implants in knee replacement: Protocol for a Bayesian network meta-analysis, analysis of joint registries and economic decision model to determine the effectiveness and cost-effectiveness of knee implants for NHS patients. The KNee Implant Prostheses Study (KNIPS)

Elsa M R Marques[1]

Jane Dennis[1]

Andrew D Beswick[1]

Julian PT Higgins[2 3]

Howard Thom[2 3]

Nicky J Welton[2 3]

Amanda Burston[1]

Linda P Hunt[1]

Michael R Whitehouse[1 3]

Ashley W Blom[1 3]

[1] Musculoskeletal Research Unit, Translational Health Sciences, Bristol Medical School, University of Bristol

[2] Population Health Sciences, Bristol Medical School, University of Bristol, Bristol

[3]National Institute for Health Research Bristol Biomedical Research Centre, University Hospitals Bristol NHS Foundation Trust and University of Bristol

e.marques@bristol.ac.uk

jane.dennis@bristol.ac.uk

andy.beswick@bristol.ac.uk

julian.higgins@bristol.ac.uk

howard.thom@bristol.ac.uk

nicky.welton@bristol.ac.uk

amanda.burston@bristol.ac.uk

l.p.hunt@bristol.ac.uk

For peer review only
For peer review only - http://bmjopen.bmj.com/site/about/guidelines.xhtml

michael.whitehouse@bristol.ac.uk

ashley.blom@bristol.ac.uk

Corresponding author: Andrew D Beswick

Musculoskeletal Research Unit

Translational Health Sciences

Bristol Medical School

University of Bristol

Learning and Research Building, Level 1

Southmead Hospital

Bristol

BS10 5NB

andy.beswick@bristol.ac.uk

Tel 01174147870

Word count: 5150

Keywords:

Arthroplasty, Replacement, Knee

Unicompartmental knee replacement

Network meta-analysis

Prostheses and implants

Cost benefit analysis

Registries

For peer review only - http://bmjopen.bmj.com/site/about/guidelines.xhtml

Abstract

Introduction

Knee replacements are highly successful for many people, but if a knee replacement fails, revision surgery is generally required. Surgeons and patients may choose from a range of implant components and combinations which make up knee replacement constructs, all with potential implications for how long a knee replacement will last. To inform surgeon and patient decisions, a comprehensive synthesis of data from randomised controlled trials is needed to evaluate the effects of different knee replacement implants on overall construct survival. Due to limited follow-up in trials, joint registry analyses are also needed to assess the long-term survival of constructs. Finally, economic modelling can identify cost-effective knee replacement constructs for different patient groups.

Methods and analysis

In this protocol we describe systematic reviews and network meta-analyses to synthesise evidence on the effectiveness of knee replacement constructs used in total and unicompartmental knee replacement, and analyses of two national joint registries to assess long-term outcomes. Knee replacement constructs are defined by bearing materials and mobility, constraint, fixation and patella resurfacing. For men and women in different age groups, we will compare the lifetime cost-effectiveness of knee replacement constructs.

Ethics and dissemination

Systematic reviews are secondary analyses of published data with no ethical approval required. We will design a common joint registry analysis plan and provide registry representatives with information for submission to research or ethics committees. The project has been assessed by the NHS REC committee and does not require ethical review.

Study findings will be disseminated to clinicians, researchers and administrators through open access articles, presentations and websites. Specific UK-based groups will be informed of results including NIHR and NICE, as well as international orthopaedic associations and charities. Effective dissemination to patients will be guided by our patient-public involvement group and include written lay summaries and infographics.

PROSPERO: CRD42019134059; CRD42019138015

Article summary

For peer review only - http://bmjopen.bmj.com/site/about/guidelines.xhtml

Strengths and limitations of the study

- Bringing evidence from multiple RCTs together in systematic reviews and meta-analysis with thorough assessments of risk of bias and confidence in the evidence ensures that health professionals have the information they need to deliver a high-quality patient experience with safe, clinically effective and cost-effective treatments.
- If there are insufficient data to build the economic model using the network of RCT studies in the literature, we will base our economic model on analyses of registry data. The joint registries that we will analyse include large numbers of patients with information on knee replacement constructs used and patient demographics such as age and sex, to enable adjustment for potential confounders.
- A limitation of the systematic review elements of our project is the likely inclusion of small RCTs with short follow up; many RCTs of knee replacement constructs report radiographic outcomes, which do not necessarily require large sample sizes and long-term follow up. Some studies we identify may not report relevant outcomes or be at high risk of bias which will reduce the number of studies and knee replacement constructs that we can include in analyses.
- A limitation of our registry analyses is that treatment choices are made for individual patients and uncontrolled confounding factors may influence outcomes.
- Exploring the effectiveness of knee replacement constructs in both RCTs and registries, and linking this information to implant and healthcare costs, will allow us to compare the lifetime cost-effectiveness of different knee replacement constructs.

For peer review only

For peer review only - http://bmjopen.bmj.com/site/about/guidelines.xhtml

Introduction

In 2018, more than 100,000 primary knee replacements were performed in the UK(1,2) and over 15,400 in Sweden, our collaborating country.(3) About 96% of operations are to treat end-stage osteoarthritis, with the majority in women (57%) at a mean age of 69 years.(1) For many people, knee replacements are highly successful and can last a person's lifetime.(4) However, when a knee replacement fails, most commonly due to loosening or wear, people may require revision surgery. Revision surgery is more complex for clinicians to perform, is difficult for patients to recover from, is associated with further complications and need for re-revisions, and is costly.

Description of the intervention

The knee joint consists of three compartments: the medial femorotibial, the lateral femorotibial and the patellofemoral, all of which can be affected by osteoarthritis with associated pain and disability. Depending on which compartments are affected, a surgeon may perform a total knee replacement (TKR) or a unicompartmental knee replacement (UKR). In TKR, both the medial and lateral compartments are replaced as well as the trochlea (groove) on the front of the femur. The patella that articulates with this may be resurfaced or not. Some surgeons favour UKR on the basis of radiographic evidence of osteoarthritis affecting a single medial or lateral compartment with estimates of patient eligibility for UKR as high as 48% of all people receiving knee replacement,(5) although actual rates of utilisation are around 10%.(1,3) Compared with TKR, UKR surgery requires a less invasive procedure, retains more bone and native ligaments, the operation has a shorter duration, is quicker for patients to recover from, and is cheaper for the NHS. However, with time, osteoarthritis can develop in the other compartments and a patient with a UKR is more than twice as likely to undergo revision than a patient who has had a TKR.(1) Revision of UKR to TKR can be complex, and may include treatment of bone defects.(6) Thus, many surgeons favour TKR as the first treatment for severe knee osteoarthritis. In the National Joint Registry for England, Wales, Northern Ireland and the Isle of Man (NJR), 87.4% of knee replacements performed in 2018 were TKR and 11% UKR.(1) In 2018 in the Swedish Knee Arthroplasty Register (SKAR), 90.0% of knee replacements were TKR and 9.6% UKR.(3)

In both TKR and UKR, the remaining cartilage and some bone in the affected portion of the joint is removed and replaced, typically with implants made of metal fixed to the bone and polyethylene bearing surfaces between the metal implants or affixed directly to the bone. Stability of the knee relies to a large degree on the ligaments in and around the knee. In

For peer review only - http://bmjopen.bmj.com/site/about/guidelines.xhtml

UKR, these are retained, in TKR, some of them are removed and their function compensated for by the implants used.

TKR construct options (Table 1)

Surgeons and patients may choose from a range of implant components and combinations which make up knee replacement constructs, all with potential implications for how long a knee replacement will last.

In a modular TKR construct, the polyethylene liner between the metal femoral and tibial components can be "fixed" to the tibial component or "mobile" with movement of the liner permitted on the tibial component. In a "monobloc" implant, the top of the tibia is replaced with a one-piece polyethylene or metal backed implant that is fixed directly to the bone.

TKR constructs can accommodate keeping the posterior cruciate ligament (cruciate retaining) or not (cruciate sacrificing or posterior stabilised). Depending on the condition of the patella and the opinion of the surgeon, the patella can be resurfaced with a polyethylene or metal-backed polyethylene implant.

Fixation of both femoral and tibial components to their respective bones is done with or without the use of cement. Alternatively, in a hybrid TKR, the tibial component is fixed with cement whilst the femoral component is uncemented. More recently, inverse (or reverse) hybrid fixation with uncemented tibial and cemented femoral components has been reported as a distinct combination.(7) Patella resurfacing can be with a cemented or cementless design.

UKR construct options (Table 1)

Depending on which compartment is affected and replaced, UKRs can be medial UKR, lateral UKR or patellofemoral UKR. As with TKR, component options are available to surgeons performing a UKR. The polyethylene liner may be fixed or mobile and implants can be fixed to bone with cement, without cement (uncemented) or using a combination (hybrid). In patellofemoral UKR, the back of the patella and trochlea of the femur are resurfaced with polyethylene and metal respectively. In rare situations, surgeons may perform multiple UKRs of different compartments at the same time.(8)

Effectiveness and cost-effectiveness of knee replacement constructs: existing knowledge

To assess the relevance of our proposed research, we performed a scoping literature search of MEDLINE and Embase in January 2020 to identify existing network meta-analyses relating to knee replacement. Most of the 33 studies we identified considered drug

For peer review only - http://bmjopen.bmj.com/site/about/guidelines.xhtml

treatments. Two systematic reviews and one protocol described the application of network meta-analysis to knee replacement constructs. One study compared resurfacing of the patella with no resurfacing and, to complete the network, with patella denervation.(9) While the authors concluded that patella resurfacing was associated with a lower rate of re-operation than not resurfacing the patella and with no benefit for pain or surgeon-assessed scores, there was no attempt to interpret results in the context of assessed risk of bias of included studies. Another systematic review of different surgeries for the treatment of osteoarthritis with network meta-analysis only included cohort studies and did not assess their risk of bias.(10) A third Cochrane review protocol describing a network meta-analysis to compare diverse surgical and medical therapies in TKR(11) was withdrawn in September 2019.(12)

In a second contemporaneous search of MEDLINE and Embase for systematic reviews, we identified six that explicitly considered the cost-effectiveness of different knee replacement constructs. The potential value of patella resurfacing was evaluated in one systematic review.(13) Over five years, the authors concluded that there was a small cost-saving when the patella was resurfaced. However, there was no consideration of risk of bias. In one systematic review, the authors concluded that the benefit of UKR compared with TKR is dependent on the economic perspective chosen, patient characteristics and the timing of outcome.(14) In three reviews, authors concluded that UKR may be a cost-effective outcome in older patients but that this was uncertain for younger patients due to lower survivorship associated with UKR constructs.(15-17) In another systematic review comparing UKR, TKR and other procedures, results presented narratively were equivocal.(18)

A further search for economic analyses and registry studies identified 13 potentially relevant studies. Registry analyses covered bearing surfaces,(19-21) monobloc tibial components,(22) patella resurfacing,(23) mobile and high flexion designs,(20) fixation,(24) patellofemoral replacement,(25) and UKR.(17,26-28) We did not find any registry studies comparable to our study comparing diverse features of knee replacement constructs. The authors of one study presented a cost-effectiveness model based on five common brands of TKR implants up to 10 years post-primary surgery.(29) Each brand studied represented a construct with cemented unconstrained components with fixed bearings, in contrast with our approach, which focuses on different constructs.

A search of PROSPERO in January 2020 identified no network meta-analyses in knee replacement comparing knee replacement constructs comparable to our planned study.

Objectives

For peer review only - http://bmjopen.bmj.com/site/about/guidelines.xhtml

The objectives of our study are to synthesise evidence from randomised controlled trials (RCTs), registries, and studies reporting quality of life and cost information, to identify:

1. the most effective and cost-effective TKR constructs for patients of different age and sex profiles
2. the most effective and cost-effective knee replacement constructs for patients of different age and sex profiles eligible for both TKR and UKR

We will achieve these objectives by conducting two systematic reviews to identify RCT evidence for each question, analysis of registries from the United Kingdom (UK) and Sweden to estimate long-term revision and mortality rates, and development of a cost-effectiveness model for each question.

The primary outcome in both systematic reviews will be revision rate and timing of revision which are key markers of construct effectiveness. Secondary outcomes will be patient-reported pain and function, and surgeon-assessed outcomes.

As the implants that make up a construct can vary in TKR and UKR, network meta-analysis with direct and indirect comparisons is an appropriate method for synthesis of data from RCTs. We will develop networks of evidence for revision (and other outcomes) at different time periods after primary TKR and UKR. These will include an initial period post primary surgery where the risk of first revision is high, a medium-term period with a lower risk of first revision and a late revision period, where risk of first revision increases.

Recognising the short follow-up in many orthopaedic RCTs, we will also analyse data on outcomes after different knee replacement constructs from two joint registries.

Outcome data from RCTs and registries, together with evidence on quality of life and construct and health service costs, will be used to inform economic decision models which will rank and estimate the cost-effectiveness of knee constructs for patients of different sex and age profiles.

Methods

Timescale

The project will collect data and perform analyses between January 2019 and December 2021.

Patient and public involvement

This proposal was developed in collaboration with the University of Bristol Musculoskeletal Research Unit patient and public involvement group. The “Patient Experience Partnership in Research” (PEP-R) group comprises nine members, most of whom have had joint

For peer review only - http://bmjopen.bmj.com/site/about/guidelines.xhtml

replacement. Meetings are facilitated by a dedicated coordinator who works in partnership with researchers to provide patient and public input into research. We met with PEP-R on two occasions for input into the development of the research proposal in 2017 and 2018, and at the start of the project in 2019. Patients told us that they were not informed about knee implant options when discussing their surgery with the consultant. PEP-R will provide ongoing support throughout the study and appropriate funding is in place. Anticipated contributions will include advice on dissemination of results, particularly plain language summaries and the use of our findings to support shared decision making.(30)

Systematic reviews and meta-analyses

The systematic reviews are registered with PROSPERO (CRD42019134059 and CRD42019138015) and a Preferred Reporting Items for Systematic Reviews and Meta-Analyses Protocols (PRISMA-P) statement(31) is provided. The research questions are formulated according to PICO(32) and review conduct will be based on methods described in the Cochrane Handbook for Systematic Reviews of Interventions.(33) Reporting will adhere to the Preferred Reporting Items for Systematic Reviews and Meta-Analyses (PRISMA) guidelines(34) and the PRISMA extension statement for reporting of systematic reviews incorporating network meta-analyses.(35)

Systematic review 1: TKR

Eligibility criteria

Types of studies

We will include RCTs.

Participants

Eligible patients will be receiving elective primary TKR (unilateral or bilateral) and aged 18 years or older. There will be a diagnosis of osteoarthritis in 50% or more of the study population.

Interventions and comparators

We will include comparisons of knee replacement constructs as summarised in Table 1. Each construct and comparator will have a combination defined by bearing mobility, constraint, fixation, bearing materials and patella resurfacing. In theory this could be 240 different combinations, but many combinations are not feasible or desirable.

The reference construct within the network meta-analysis will be the most widely used TKR construct which has cemented, cruciate retaining, fixed bearing implant components with metal on polyethylene bearing materials.

For peer review only - http://bmjopen.bmj.com/site/about/guidelines.xhtml

For peer review only

Types of outcome measures

The primary outcome of the systematic reviews will be first revision surgery after primary TKR at any time from primary surgery. We will collect data at each time point reported in articles and, for revision outcome, extract data from published Kaplan-Meier plots if available.(36)

Secondary outcomes will include: further revision surgeries; deaths; reason for revision; patient-reported outcomes such as the Oxford Knee Score (OKS)(37) and Western Ontario and McMaster Universities Arthritis Index (WOMAC);(38) clinician-assessed measures including the American Knee Society Score (KSS)(39) and Hospital for Special Surgery (HSS) score;(40) and quality of life indices such as the EuroQol questionnaire.(41)

We will collect information on surgical complications and major adverse events including infection and deep vein thrombosis, and on hospital readmissions.

Search strategy

Online databases to be searched from inception are MEDLINE, Embase, and PsycINFO on Ovid, CINAHL on EBSCOhost and the Cochrane Library. Searches of PsycINFO and CINAHL are unlikely to identify further RCTs but are routinely searched in our department. Online databases will be searched so that searches are up to date in October 2020. The search strategy for application in MEDLINE shown in Box 1 will be tailored to each database. As well as RCTs, our searches will identify systematic reviews which will be screened for RCTs as will reference lists of relevant RCTs. Citations of key articles will be tracked in Web of Science. We will identify clinical trial records in the Cochrane Library and check them for full publication. Should no further publication be identified, we will contact authors for details of progress and study results.

No language restrictions will be applied to searches or study inclusion. Translations will be made by colleagues or professional translators when required. Studies that are unobtainable through our library (including inter-library loans) and via author contact will be excluded. If studies have not reported any follow-up data or reporting is limited regarding outcomes and study conduct, including conference abstracts, we will contact authors for appropriate data. If not available, these studies will be excluded. A summary table of excluded RCTs will be produced.

Data management

Selection of studies

Records will be imported into Endnote X9 (Clarivate Analytics). An initial screen by one reviewer will exclude clearly irrelevant articles. Subsequently, abstracts and full articles will

For peer review only - http://bmjopen.bmj.com/site/about/guidelines.xhtml

be screened independently in Covidence® by two reviewers and reasons for exclusion recorded. Discrepancies between reviewers will be resolved by consensus with involvement of an orthopaedic surgeon or methodologist. Authors will be contacted by email to confirm eligibility if necessary. Multiple reports of RCTs will be grouped together as a single study defined by a key publication with follow-up data. Notices of errata and retractions will be sought.

Data extraction

After piloting of forms, data will be extracted into Covidence®, Microsoft Access or Excel by one reviewer. Data to be extracted will be: country; dates of recruitment; participant characteristics including indication, age and sex; inclusion and exclusion criteria; knee replacement constructs defined by bearing mobility, constraint, fixation, bearing materials and patella resurfacing; other surgical methods including approach, alignment, computer navigation and robot assistance; rehabilitation regime; study setting to include number of surgeons and centres; sponsorship; follow-up intervals; outcome data; and information to assess risk of bias. For the categorical outcome of revision, we will collect the number of events recorded up to a particular follow up or mean follow-up time. For continuous outcomes, we will extract means and standard deviations or, if unavailable, make estimates based on medians, interquartile ranges and ranges.(33) We will contact authors of eligible studies by email to maximise available detail concerning risk of bias, effect estimates and measures of variability for the outcomes of interest.

Depending on the number of studies identified, study characteristics will be fully extracted or checked against source material by a second reviewer. Outcome data will be extracted independently by two reviewers.

Risk of bias assessment

Risk of bias of eligible RCTs will be assessed with the revised Cochrane tool (RoB 2).(42) Protocols will be sought to identify evidence of selective outcome reporting. Assessments will be made for all RCTs by two reviewers working independently with disagreements resolved with other members of the review team. Risk of bias for individual studies will be assessed as low, some concerns, or high risk of bias. In surgical trials, key concerns relate to deviations from intended interventions (e.g. large proportion of patients not receiving randomised allocation) and bias due to missing outcome data (e.g. high or unequal loss to follow up). Aspects of risk of bias will be considered in both meta-analysis and narrative synthesis. Data analysis in all meta-analyses will exclude studies assessed at high risk of bias.

Data analysis

For peer review only - http://bmjopen.bmj.com/site/about/guidelines.xhtml

Pairwise meta-analysis

Data synthesis for the different knee replacement constructs defined by constraint, mobility, fixation, and material will start with a tabulation of study details and narrative synthesis. The unit of analysis will be the knee.

Data will be analysed in three different time periods with the cut-offs of the period time-points varied in sensitivity analysis. Initially, the periods at risk will be: “early stage failure” when the construct fails within the first three years following knee replacement; “medium stage failure” when the construct survives the early period but fails within ten years from the primary surgery; and “late-stage failure” when the construct first fails ten or more years after the primary surgery.

Data analyses will start with pairwise meta-analyses for each comparison of knee replacement constructs. The effect measure for revision will be the hazard rate ratio. Patient-reported and surgeon-assessed outcomes are likely to be continuous outcome scores, which will be analysed using difference in mean or standardised difference in mean scores.

If the pairwise meta-analyses include ten or more RCTs we will produce funnel plots and check for asymmetry which may reflect publication bias.(43) If asymmetry is noted we will perform sensitivity analyses with exclusion of small studies, or conduct meta-regression.(33)

Network meta-analysis

We will construct a network of studies comparing different knee replacement constructs and use network meta-analysis to estimate treatment effects informed by both direct comparisons within trials and indirect comparisons across trials. Hazard ratios over the three separate time periods (early, medium, and late-stage failure) will be assumed piecewise constant but the hazard ratio of the latter two periods will be normally distributed around that of the prior period.(44) These methods will broadly follow those we have used previously.(45,46) We do not aim to extrapolate outside of the follow-up time of RCTs as this would rely on assumptions about the parametric form; as explained below we will use registry data for long term rates, in line with recommendations of the NICE Decision Support Unit.(47)

Network plots will be generated for each outcome to illustrate which interventions are compared directly and indirectly, and the strength of the direct and indirect evidence including the number of studies and patients available for each direct comparison. Network meta-analysis will be implemented in a Bayesian framework using OpenBUGS software (v3.2.3).(48) We will adapt code developed for our hip replacement surgery network meta-analysis,(46) which itself was adapted from published code.(44) In our main analysis, each

For peer review only - http://bmjopen.bmj.com/site/about/guidelines.xhtml

construct will be considered a separate intervention forming a distinct node in the network of evidence. However, this may result in a disconnected network or imprecise effect estimates. We will therefore also fit a component network meta-analysis model which assumes additivity of the components of each construct.(49) The additivity assumption will be assessed by comparing model fit between the model where each construct is distinct and the additive model.

Heterogeneity and subgroup analyses

We aim to fit both fixed and random effects models where possible, and assess heterogeneity by inspection of the between studies standard deviation and comparison of model fit between the fixed and random effects models.(33,50) However, we anticipate that there may be too few replications of individual comparisons to fit a random effects model, in which case we will implement an informative, evidence-based prior distribution for the heterogeneity variance.(51) Model fit is measured by the posterior mean residual deviance (which we expect to be similar to the number of data points), and the deviance information criteria(52) (which penalises fit with a measure of model complexity), with a preference for models where these measures are smaller (where differences of at least three are considered meaningful). If data allow, we will investigate whether revision rates vary according to participant, knee replacement construct and trial characteristics (including concerns relating to risk of bias), and surgical methods including approach, alignment, computer navigation and robot assistance.

If we observe a high level of heterogeneity and cannot explain it by patient, study or surgical factors, results from the random-effects meta-analysis will be reported. If no evidence of heterogeneity is found, we will report results from the fixed effect model.

We will assess consistency between direct and indirect evidence by comparing the fit of the consistency model with the fit of an inconsistency model (the unrelated mean effects model), which relaxes the consistency assumption. Model fit statistics will be compared and the contribution to the posterior mean residual deviance for each study will be plotted for the consistency model against the inconsistency model, to identify any particular studies contributing to inconsistency.(50)

Confidence in the evidence

Our confidence in the evidence for each outcome for each intervention will be assessed using an extension of the Grading of Recommendations Assessment, Development, and Evaluation (GRADE) framework developed for network meta-analysis and implemented in the CINeMA tool.(53,54)

For peer review only - http://bmjopen.bmj.com/site/about/guidelines.xhtml

Systematic review 2: UKR and TKR

The systematic review and meta-analysis methods used comparing UKR with TKR in Review 2 will be similar to those used in Review 1.

Eligibility criteria

Types of studies

We will include RCTs.

Participants

Eligible patients will be as described in Review 1 but will have been assessed as eligible for both an elective UKR and an elective TKR.

Interventions and comparators

The knee replacement constructs will include all components used in medial, lateral and patellofemoral UKR and in TKR.

To complete the network of studies in Review 2, we will include studies comparing:

2a) different types of UKR constructs

2b) UKR with TKR constructs

2c) TKR constructs for patients eligible for UKR

Ascertaining the eligibility of trial participants for c) will be challenging due to paucity of information on patient characteristics described at trial level (e.g. number of symptomatic compartments) and time trends in clinical practice. Clinicians in our team will assess which trials may “possibly include” patients potentially eligible for UKR in a gradient up to studies which “definitely exclude” patients eligible for UKR.

Types of outcome measures

Outcome measures will be as described in Review 1.

Search strategy

The search in Review 1 includes terms for UKR and thus will identify relevant studies. Methods used will mirror those of Review 1.

Data management

Management of study information, screening and data extraction will be as described in Review 1. Data extraction will additionally include details of whether the UKR is for treatment of osteoarthritis in the medial, lateral or patellofemoral compartment.

For peer review only - http://bmjopen.bmj.com/site/about/guidelines.xhtml

For peer review only

Risk of bias assessment

Assessment of risk of bias will use methods described in Review 1.

Data analysis

For pairwise and network meta-analyses, we plan main analyses restricted to studies 2a) comparing different UKR constructs and 2b) comparing UKR and TKR constructs. Secondary analyses 2c) will also include comparisons of different TKR constructs in patients eligible for UKR.

For Review 2a), the reference construct will be the most widely used cemented mobile bearing. In Reviews 2b) and 2c) the reference construct within the network meta-analysis will be the TKR combination of cemented, cruciate retaining, fixed bearing implant components with metal on polyethylene bearing materials.

The methods for analysis will otherwise be as in Review 1.

Joint registry analysis

Joint registries

In our previous research(55) we have established close links between the National Joint Registry for England, Wales, Northern Ireland and the Isle of Man (NJR) and the Swedish Hip Arthroplasty Register (SHAR).(56) This was a successful collaboration and our analyses benefitted from the use of similar data collection and management in the two databases and the long follow-up of patients in Sweden. Recognising this, we have established links with the Swedish Knee Arthroplasty Register (SKAR).(57)

Data collection in the NJR commenced on April 1st, 2003 and includes 1,193,830 primary knee replacements with verifiable patient data up to 31st December 2018.(1) In 2020, up to 17 years of patient follow-up data is available for analysis.

SKAR was established in 1975 and includes patient identifier numbers which allows tracking of other healthcare use.(58) More complete data with information required for our study has been collected since 1989. Thus, in 2020, the registry includes up to 30 years of patient follow-up data.

Eligibility criteria

Patient group

Patients included in analyses will be aged 18 years or over, undergoing primary TKR or UKR for osteoarthritis, with valid data on age, sex and construct characteristics.

Interventions and comparisons

For peer review only - http://bmjopen.bmj.com/site/about/guidelines.xhtml

Knee replacement constructs for comparison will be characterised by bearing mobility, constraint, fixation and bearing materials. Patella resurfacing is recorded in the NJR but is infrequently used in Sweden. We will exclude patients receiving patellofemoral and bicompartmental knee replacement as they are generally used in a selected patient group.

The reference construct will be TKR with cemented, cruciate retaining, fixed bearing implant components with metal on polyethylene bearing materials.

Outcomes

Outcomes for analyses will be time to first, second and subsequent revision surgeries, and death after primary surgery.

Statistical analysis

We will prepare a joint statistical analysis plan for analysis of NJR and SKAR data for our research questions. The analysis plan will include: definitions of primary surgery, knee replacement constructs and techniques, follow-up time frames and age groups; statistical methods including handling of missing data; and patient characteristics for a possible subgroup of TKR patients eligible for UKR. We will estimate hazard rates of first and second revision, hazard ratios between implants, and the effect of covariates (e.g. age, gender, time since primary surgery, time since first revision) on these parameters.

The analyses will be performed using STATA software. Estimates from the SKAR population will be calibrated to the NJR population using a range of calibration models implemented in OpenBUGs software as in our previous study.(55) These calibrations will model some difference between SKAR and NJR estimates of hazard rates and hazard ratios which is either fixed, random, or independent over time periods.

Cost-effectiveness analysis

Economic decision models

RCT and registry data will be combined with cost data and utility weight estimates to inform a probabilistic lifetime decision economic model to determine the relative cost-effectiveness from an NHS and social services perspective of 1) TKR constructs and 2) TKR and UKR constructs for patients eligible for both. Results will be stratified by sex and age-group. We will limit knee replacement constructs to those available for clinical use in the NHS.

We will calculate expected costs and quality-adjusted life years (QALYs), discounted at 3.5% per annum using a fully probabilistic analysis, which reflects parameter uncertainty in the sampled distributions, and simulating some number of iterations. We will first use 10,000 iterations and check if it is sufficient for the mean and standard deviation of the cost and

For peer review only - http://bmjopen.bmj.com/site/about/guidelines.xhtml

For peer review only

QALYs to converge; more simulations will be employed if needed. The reference construct for both models will be TKR with cemented, cruciate retaining, fixed bearing implant components with metal on polyethylene bearing materials.

The economic models will be implemented in the open source statistical software R.(59) The R software has well documented advantages of speed, flexibility, and transparency over other software such as Excel.(60) The same models will be fitted to both decision populations. Two model structures will be explored (full details are included in Supplementary material). First is a cohort Markov multistate model with health states representing time since primary surgery, post first revision, post second or higher revision, and death.(61) States for time since primary will correspond to <3 years, ≥3 years but <10 years, and ≥10 years post primary. The post 1st revision states will correspond to early (<3 years), middle (≥3 years but <10 years), and late (≥10 years) revision. Probabilities of 1st revision will depend on the time since primary while probabilities of 2nd revision will depend on whether the 1st revision was early, middle, or late. Probabilities of 1st revision for each construct and time period and for 2nd revision will be calculated using area-under-the-curve of estimated hazard rates.(62) For 1st revision, rates for the reference construct will come from the NJR/SKAR analysis. Rates of 1st revision for other constructs will be calculated by applying time-period specific hazard ratios from the network meta-analysis or, if RCT and thus network meta-analysis data are not available, NJR/SKAR data.

The second model structure we will use is an individual level continuous time semi-Markov model.(63) We aim to employ the 'hesim' extension to R to implement this model.(64) States will correspond to no revision, post 1st revision, post 2nd revision, and death. Transition rates of 1st revision will depend on time since primary surgery and patient characteristics (e.g. age and gender). Rates of 2nd revision will depend on time from primary to 1st revision and time since 1st revision. Model parameters will be estimated using the same data as for the cohort Markov model, but without a conversion to probabilities. NJR/SKAR will provide rates of 1st revision for our reference construct. These will be adjusted to other constructs using hazard ratios from the network meta-analysis or, if RCT and thus network meta-analysis data are not available for the construct or time period, NJR/SKAR estimates of hazard ratios. NJR/SKAR will provide rates of 2nd revision, which will be assumed common across constructs. This is a refinement of the cohort Markov multistate model and captures the main deviations from a Markov model in the disease area. While results are expected to be similar, our base case will be the individual level continuous time semi-Markov model as it places fewer restrictions on timings of events.

We will conduct a literature search to identify the best data source to derive utility weight estimates stratified by age and sex for use in the models. A potential source is the UK

For peer review only - http://bmjopen.bmj.com/site/about/guidelines.xhtml

Patient Reported Outcome Measures (PROMs) dataset for patients with knee replacement.(65) The PROMs dataset includes EQ-5D-3L data at six months post-surgery for patients over 40 years old.

Construct prices in GB pounds will be estimated from a national database of NHS trusts providing implant procurement prices to the NJR: the NJR INFORM Implant price-benchmarking database.(66) Primary and revision surgeries, and follow-up healthcare costs will be obtained from national tariffs.(67) We will search the literature for published estimates of primary and secondary care follow-up costs from cost-effectiveness analyses in RCTs of knee replacement. The validity of these costs in the context of our implant construct comparisons will be assessed by clinicians in our team.

Cost-effectiveness will be estimated using the mean incremental net monetary benefit statistic (INMB) for each knee replacement construct compared to the reference construct, a willingness-to-pay threshold of £20,000 per QALY.(68) The construct with the highest INMB is the most cost-effective for each patient subgroup. With cost-effectiveness acceptability curves we will show how the probability of knee replacement constructs being most cost-effective varies as willingness-to-pay thresholds change.

In sensitivity analyses we will assess the robustness of the results to changes in key parameters and assumptions. These will involve variation of: time periods over which event hazards are assumed constant; estimations of transition probabilities (network meta-analysis only, registries only, combination of both); model structure to include different health states; costs of constructs; time in theatre to perform different surgeries; revision TKR costs; and other follow-up costs.

Ethics and dissemination

The systematic reviews will not require ethical approval as we are undertaking secondary analyses of published data. For the analyses of joint registry data, we will design a common analysis plan and provide representatives of the NJR and SKAR with information required for submission to their respective ethics committees. The project has been assessed with the NHS REC committee and does not require its ethical review.

We expect the results from our study to provide direct patient benefit through better information available for clinical surgical teams performing TKRs, which will influence the commissioning of knee replacement constructs used at NHS trusts in the UK. We will publish our effectiveness and cost-effectiveness findings in two separate peer-reviewed journal articles in high impact open access journals. The findings of our studies will also be

For peer review only - http://bmjopen.bmj.com/site/about/guidelines.xhtml

disseminated to the NHS, NJR, clinicians, societies, charities, patients and the general public via presentations, reports and websites.

Discussion

Surgeons and patients are faced with many construct and technique options for use in knee replacement. Patients in our PPI group told us that they were not informed about surgical choices when discussing surgery with their consultant. In a stakeholders meeting, knee surgeons advised us that they chose knee replacement constructs based on their preferences, surgical skills, availability of implants from manufacturers and available research evidence. There is a need for a comprehensive synthesis of evidence to inform this choice.

Given the large number of knee replacements performed annually and stringent NHS budgets, it is important to determine which knee replacement constructs provide the best outcomes for patients at lowest cost to the NHS. Ideally, all constructs would be compared in an RCT with long-term follow-up and enough statistical power to estimate differences in revision rates between constructs. However, it would be difficult to compare large numbers of constructs in an RCT and fund long-term follow-up.

We suggest that a more practical and efficient approach is to use all available evidence in published head-to-head RCTs to obtain treatment effect estimates for knee replacement constructs. We will complement these with analyses of large longitudinal national registries of surgical and patient data with long-term follow-up. Both estimates will be used to produce economic decision models which will rank and estimate the cost-effectiveness of knee replacement constructs.

Author statement

All authors contributed to the concept and design of the study. EMRM, HT and ADB drafted the article and JD, JPTH, NJW, AB, LPH, MRW and AWB revised it critically for important intellectual content. EMRM and ADB take responsibility for the integrity of the work as a whole, from inception to finished article.

Data statement

All relevant data identified in the systematic review will be included in the published final report and supplementary material.

Funding source

For peer review only - http://bmjopen.bmj.com/site/about/guidelines.xhtml

This study is funded by the National Institute for Health Research (NIHR) Research for Patient Benefit programme (project reference PB-PG-1013-32010). The views expressed are those of the authors and not necessarily those of the NIHR or the Department of Health and Social Care.

The funder had no role in the design of the study and collection, analysis, and interpretation of data and in writing the manuscript. The researchers were independent from funders and all authors had full access to all of the data in the study and can take responsibility for the integrity of the data and the accuracy of the data analysis. Funding for Review 2 is applied for.

Competing interests

All authors declare no conflicts of interest.

For peer review only

References

1. National Joint Registry for England, Wales, Northern Ireland and the Isle of Man: 16th Annual report. Hemel Hempstead: NJR Centre 2019.

2. Scottish Arthroplasty Project. Annual report 2019. Edinburgh: NHS National Services Scotland; 2019.

3. Swedish Knee Arthroplasty Register: Annual report 2019. Lund: Skåne University Hospital; 2019.

4. Evans JT, Walker RW, Evans JP, Blom AW, Sayers A, Whitehouse MR. How long does a knee replacement last? A systematic review and meta-analysis of case series and national registry reports with more than 15 years of follow-up. Lancet. 2019;393(10172):655-63.

5. Willis-Owen CA, Brust K, Alsop H, Miraldo M, Cobb JP. Unicondylar knee arthroplasty in the UK National Health Service: An analysis of candidacy, outcome and cost efficacy. Knee. 2009;16(6):473-8.

6. Wynn Jones H, Chan W, Harrison T, Smith TO, Masonda P, Walton NP. Revision of medial Oxford unicompartmental knee replacement to a total knee replacement: Similar to a primary? Knee. 2012;19(4):339-43.

7. Nieneläinen MJ, Mäkelä KT, Robertsson O, W-Dahl A, Furnes O, Fenstad AM, et al. The effect of fixation type on the survivorship of contemporary total knee arthroplasty in patients younger than 65 years of age: a register-based study of 115,177 knees in the Nordic Arthroplasty Register Association (NARA) 2000-2016. Acta Orthop. 2020;91(2):184-90.

8. Ma J-x, He W-w, Kuang M-j, Sun L, Lu B, Wang Y, et al. Efficacy of bicompartmental knee arthroplasty (BKA) for bicompartmental knee osteoarthritis: A meta analysis. Int J Surg. 2017;46:53-60.

9. Arirachakaran A, Sangkaew C, Kongtharvonskul J. Patellofemoral resurfacing and patellar denervation in primary total knee arthroplasty. Knee Surg Sports Traumatol Arthrosc. 2015;23(6):1770-81.

10. Liu CY, Li CD, Wang L, Ren S, Yu FB, Li JG, et al. Function scores of different surgeries in the treatment of knee osteoarthritis: A PRISMA-compliant systematic review and network-meta analysis. Medicine. 2018;97(21):e10828.

11. Singh JA, Tugwell P, Zanoli G, Wells GA. Total joint replacement surgery for knee osteoarthritis and other non-traumatic diseases: A network meta-analysis. Cochrane Database Syst Rev. 2015;2015 (6)(CD011765).

12. Singh JA, Tugwell P, Zanoli G, Wells GA. Total joint replacement surgery for knee osteoarthritis and other non-traumatic diseases: a network meta-analysis. Cochrane Database Syst Rev. 2019(9).

For peer review only - http://bmjopen.bmj.com/site/about/guidelines.xhtml

13. Meijer KA, Dasa V. Is resurfacing the patella cheaper? An economic analysis of evidence based medicine on patellar resurfacing. Knee. 2015;22(2):136-41.
14. Daigle ME, Weinstein AM, Katz JN, Losina E. The cost-effectiveness of total joint arthroplasty: a systematic review of published literature. Best Pract Res Clin Rheumatol. 2012;26(5):649-58.
15. Burn E, Liddle AD, Hamilton TW, Pai S, Pandit HG, Murray DW, et al. Choosing between unicompartmental and total knee replacement: What can economic evaluations tell us? A systematic review. Pharmacoeconom Open. 2017;1(4):241-53.
16. Chawla H, Ghomrawi HM, van der List JP, Eggman AA, Zuiderbaan HA, Pearle AD. Establishing age-specific cost-effective annual revision rates for unicompartmental knee arthroplasty: A meta-analysis. J Arthroplasty. 2017;32(1):326-35.
17. Smith WB, Steinberg J, Scholtes S, McNamara IR. Medial compartment knee osteoarthritis: age-stratified cost-effectiveness of total knee arthroplasty, unicompartmental knee arthroplasty, and high tibial osteotomy. Knee Surg Sports Traumatol Arthrosc. 2017;25(3):924-33.
18. Kamaruzaman H, Kinghorn P, Oppong R. Cost-effectiveness of surgical interventions for the management of osteoarthritis: a systematic review of the literature. BMC Musculoskelet Disord. 2017;18(1):183.
19. Fennema P, Heyse TJ, Uyl-de Groot CA. Cost-effectiveness and clinical implications of advanced bearings in total knee arthroplasty: a long-term modeling analysis. Int J Technol Assess Health Care. 2014;30(2):218-25.
20. Gioe T, Sharma A, Tatman P, Mehle S. Do "premium" joint implants add value? Analysis of high cost joint implants in a community registry (Provisional abstract). Clin Orthop Relat Res. 2011;469(1):48-54.
21. Partridge TCJ, Baker PN, Jameson SS, Mason J, Reed MR, Deehan DJ. Conventional versus highly cross-linked polyethylene in primary total knee replacement: A comparison of revision rates using data from the National Joint Registry for England, Wales, and Northern Ireland J Bone Joint Surg Am. 2019;102(2):119-27.
22. Gioe TJ, Sinner P, Mehle S, Ma W, Killeen KK. Excellent survival of all-polyethylene tibial components in a community joint registry. Clin Orthop Relat Res. 2007;464:88-92.
23. Weeks CA, Marsh JD, MacDonald SJ, Graves S, Vasarhelyi EM. Patellar resurfacing in total knee arthroplasty: A cost-effectiveness analysis. J Arthroplasty. 2018;33(11):3412-5.
24. Manoli A, Markel JF, Pizzimenti NM, Markel DC. Early results of a modern uncemented total knee arthroplasty system. Orthopedics. 2019;42(6):355-60.
25. Chawla H, Nwachukwu BU, van der List JP, Eggman AA, Pearle AD, Ghomrawi HM. Cost effectiveness of patellofemoral versus total knee arthroplasty in younger patients. Bone Joint J. 2017;99-B(8):1028-36.

For peer review only - http://bmjopen.bmj.com/site/about/guidelines.xhtml

26. Peersman G, Jak W, Vandenlangenbergh T, Jans C, Cartier P, Fennema P. Cost-effectiveness of unicondylar versus total knee arthroplasty: a Markov model analysis. Knee. 2014;21 Suppl 1:S37-42.

27. Burn E, Liddle AD, Hamilton TW, Judge A, Pandit HG, Murray DW, et al. Cost-effectiveness of unicompartmental compared with total knee replacement: a population-based study using data from the National Joint Registry for England and Wales. BMJ Open. 2018;8(4):e020977.

28. Kolovos S, Strauss VY, Prats-Uribe A, Prieto-Alhambra D, Pinedo-Villanueva R. Hospital costs and outcomes of unicompartemental compared to total knee replacement for patients with multiple comorbidities: A population-based study. Value Health. 2019;22 (Supplement 3):S921.

29. Pennington M, Grieve R, Black N, van der Meulen JH. cost-effectiveness of five commonly used prosthesis brands for total knee replacement in the UK: A study using the NJR dataset. PLoS One. 2016;11(3):e0150074.

30. Shared physician-patient responsibilities. Position statement 1182. Rosemont, Illinois: American Academy of Orthopaedic Surgeons; 2016.

31. Moher D, Shamseer L, Clarke M, Ghersi D, Liberati A, Petticrew M, et al. Preferred reporting items for systematic review and meta-analysis protocols (PRISMA-P) 2015 statement. Syst Rev. 2015;4:1.

32. Richardson WS, Wilson MC, Nishikawa J, Hayward RS. The well-built clinical question: a key to evidence-based decisions. ACP J Club. 1995;123(3):A12-3.

33. Higgins JPT, Thomas J, Chandler J, Cumpston M, Li T, Page MJ, et al. Cochrane Handbook for Systematic Reviews of Interventions. 2nd Edition. Chichester (UK): John Wiley & Sons; 2019.

34. Moher D, Liberati A, Tetzlaff J, Altman DG. Preferred reporting items for systematic reviews and meta-analyses: the PRISMA statement. BMJ. 2009;339:b2535.

35. Hutton B, Salanti G, Caldwell DM, Chaimani A, Schmid CH, Cameron C, et al. The PRISMA extension statement for reporting of systematic reviews incorporating network meta-analyses of health care interventions: Checklist and explanations. PRISMA extension for network meta-analysis. Ann Intern Med. 2015;162(11):777-84.

36. Guyot P, Ades AE, Ouwens MJ, Welton NJ. Enhanced secondary analysis of survival data: reconstructing the data from published Kaplan-Meier survival curves. BMC Med Res Methodol. 2012;12:9.

37. Murray D, Fitzpatrick R, Rogers K, Pandit H, Beard D, Carr A, et al. The use of the Oxford hip and knee scores. J Bone Joint Surg Br. 2007;89-B(8):1010-4.

38. Bellamy N, Buchanan WW, Goldsmith C, Campbell J, Stitt L. Validation study of WOMAC: A health status instrument for measuring clinically important patient relevant

For peer review only - http://bmjopen.bmj.com/site/about/guidelines.xhtml

outcomes to antirheumatic drug therapy in patients with osteoarthritis of the hip or knee. J Rheumatol. 1989;15:1833-40.

39. Insall JN, Dorr LD, Scott RD, Scott WN. Rationale of the Knee Society clinical rating system. Clin Orthop Relat Res. 1989(248):13-4.

40. Insall JN, Ranawat CS, Aglietti P, Shine J. A comparison of four models of total knee-replacement prostheses. J Bone Joint Surg Am. 1976;58(6):754-65.

41. Brooks R. EuroQol: the current state of play. Health Policy. 1996;37(1):53-72.

42. Sterne JAC, Savović J, Page MJ, Elbers RG, Blencowe NS, Boutron I, et al. RoB 2: a revised tool for assessing risk of bias in randomised trials BMJ 2019;366:l4898.

43. Sterne JA, Sutton AJ, Ioannidis JP, Terrin N, Jones DR, Lau J, et al. Recommendations for examining and interpreting funnel plot asymmetry in meta-analyses of randomised controlled trials. BMJ. 2011;343:d4002.

44. Lu G, Ades AE, Sutton AJ, Cooper NJ, Briggs AH, Caldwell DM. Meta-analysis of mixed treatment comparisons at multiple follow-up times. Stat Med. 2007;26(20):3681-99.

45. Marques EMR, Humphriss R, Welton NJ, Higgins JPT, Hollingworth W, Lopez-Lopez JA, et al. The choice between hip prosthetic bearing surfaces in total hip replacement: a protocol for a systematic review and network meta-analysis. Syst Rev. 2016;5(1):19.

46. Lopez-Lopez JA, Humphriss RL, Beswick AD, Thom HHZ, Hunt LP, Burston A, et al. Choice of implant combinations in total hip replacement: systematic review and network meta-analysis. BMJ. 2017;359:j4651.

47. Latimer N. NICE DSU technical support document 14: Survival analysis for economic evaluations alongside clinical trials - extrapolation with patient-level data. Sheffield, UK: School of Health and Related Research, University of Sheffield; 2013.

48. Lunn D, Spiegelhalter D, Thomas A, Best N. The BUGS project: Evolution, critique and future directions. Stat Med. 2009;28(25):3049-67.

49. Welton NJ, Caldwell DM, Adamopoulos E, Vedhara K. Mixed treatment comparison meta-analysis of complex interventions: psychological interventions in coronary heart disease. Am J Epidemiol. 2009;169(9):1158-65.

50. Dias S, Ades AE, Welton NJ, Jansen JP, Sutton AJ. Network meta-analysis for comparative effectiveness research. Hoboken NJ: Wiley; 2018.

51. Turner RM, Davey J, Clarke MJ, Thompson SG, Higgins JP. Predicting the extent of heterogeneity in meta-analysis, using empirical data from the Cochrane Database of Systematic Reviews. Int J Epidemiol. 2012;41(3):818-27.

52. Spiegelhalter DJ, Best NG, Carlin BP, Van Der Linde A. Bayesian measures of model complexity and fit. J R Stat Soc Series B Stat Methodol. 2002;64(4):583-639.

For peer review only - http://bmjopen.bmj.com/site/about/guidelines.xhtml

53. Nikolakopoulou A, Higgins JPT, Papakonstantinou T, Chaimani A, Del Giovane C, Egger M, et al. CINeMA: An approach for assessing confidence in the results of a network meta-analysis. PLOS Medicine. 2020;17(4):e1003082.
54. Papakonstantinou T, Nikolakopoulou A, Higgins JPT, Egger M, Salanti G. CINeMA: Software for semiautomated assessment of the confidence in the results of network meta-analysis. Campbell Syst Rev. 2020;16(1):e1080.
55. Fawsitt CG, Thom HHZ, Hunt LP, Nemes S, Blom AW, Welton NJ, et al. Choice of prosthetic implant combinations in total hip replacement: Cost-effectiveness analysis using UK and Swedish hip joint registries data. Value Health. 2018;22:303-12.
56. Malchau H, Herberts P, Eisler T, Garellick G, Soderman P. The Swedish Total Hip Replacement Register. J Bone Joint Surg Am. 2002;84-A Suppl 2:2-20.
57. Knutson K, Lewold S, Robertsson O, Lidgren L. The Swedish knee arthroplasty register: A nation-wide study of 30,003 knees 1976-1992. Acta Orthop Scand. 1994;65(4):375-86.
58. Robertsson O, Ranstam J, Sundberg M, W-Dahl A, Lidgren L. The Swedish Knee Arthroplasty Register: a review. Bone Joint Res. 2014;3(7):217-22.
59. R Core Team. R: A language and environment for statistical computing. Vienna: Foundation for Statistical Computing; 2015.
60. Incerti D, Thom H, Baio G, Jansen JP. R you still using Excel? The advantages of modern software tools for health technology assessment. Value Health. 2019;22(5):575-9.
61. Siebert U, Alagoz O, Bayoumi AM, Jahn B, Owens DK, Cohen DJ, et al. State-transition modeling: a report of the ISPOR-SMDM Modeling Good Research Practices Task Force--3. Value Health. 2012;15(6):812-20.
62. Kalbfleisch JD, Prentice RL. The statistical analysis of failure time data. 2nd ed. Hoboken, NJ: John Wiley and sons; 2002.
63. Williams C, Lewsey JD, Briggs AH, Mackay DF. Cost-effectiveness analysis in R using a multi-state modeling survival analysis framework: A tutorial. Med Decis Making. 2017;37(4):340-52.
64. Incerti D, Jansen JP, R Core Team. Health-economic simulation modeling and decision analysis 2020 [Available from: https://cran.r-project.org/web/packages/hesim/hesim.pdf.
65. Finalised Patient Reported Outcome Measures (PROMs) in England for Hip and Knee Replacement procedures (April 2017 to March 2018) Leeds: NHS Digital; 2019 [Available from: https://digital.nhs.uk/data-and-information/publications/statistical/patient-reported-outcome-measures-proms/hip-and-knee-replacement-procedures---april-2017-to-march-2018.
66. Highlights: Economic model & price benchmarking. Hemel Hempstead: National Joint Registry for England, Wales, Northern Ireland and the Isle of Man; 2016.
67. NHS. NHS Reference Costs 2014 to 2015. London: Department of Health; 2015.

For peer review only - http://bmjopen.bmj.com/site/about/guidelines.xhtml

68. Guide to the methods of technology appraisal 2013. Process and methods. London: National Institute for Health and Care Excellence; 2013.

For peer review only

For peer review only - http://bmjopen.bmj.com/site/about/guidelines.xhtml

Table 1. Knee construct options

**Total knee replacement**					
Bearing mobility	Fixed bearing	Mobile bearing			
Constraint	Cruciate retaining	Posterior stabilised	Bi-cruciate stabilised	Constrained condylar	Hinged
Fixation	Cemented	Uncemented	Hybrid	Inverse hybrid	
Bearing materials	Metal bearing on conventional or highly cross-linked polyethylene tray	Metal femoral component on all-polyethylene or metal tibial component (monobloc)	Other materials (ceramic bearings, ceramicized metals)	Specialised or customised implants	
Patella resurfacing	Patella resurfacing cemented	Patella resurfacing uncemented	No patella resurfacing		
**Unicompartmental knee replacement (medial or lateral)**					
Bearing mobility	Fixed bearings	Mobile bearing			
Constraint	Cruciate retaining				
Fixation	Cemented	Uncemented	Hybrid		
Bearing materials	Metal on polyethylene	Metal femoral component (monobloc)			

For peer review only

Box 1. Search strategy as applied in MEDLINE

1	controlled clinical trial.pt.
2	randomized controlled trial.pt.
3	clinical trials as topic/
4	(randomi#ed or randomi#ation or randomi#ing).ti,ab,kf.
5	(RCT or "at random" or (random* adj3 (administ* or allocat* or assign* or class* or cluster or crossover or cross-over or control* or determine* or divide* or division or distribut* or expose* or fashion or number* or place* or pragmatic or quasi or recruit* or split or subsitut* or treat*))).ti,ab,kf.
6	placebo.ab,ti,kf.
7	trial.ti.
8	(control* adj3 group*).ab.
9	(control* and (trial or study or group*) and (waitlist* or wait* list* or ((treatment or care) adj2 usual))).ti,ab,kf.
10	((single or double or triple or treble) adj2 (blind* or mask* or dummy)).ti,ab,kf.
11	double-blind method/ or random allocation/ or single-blind method/
12	or/1-11
13	(systematic or structured or evidence or trials or studies).ti. and ((review or overview or look or examination or update* or summary).ti. or review.pt.)
14	(0266-4623 or 1469-493X or 1366-5278 or 1530-440X or 2046-4053).is.
15	meta-analysis.pt. or (meta-analys* or meta analys* or metaanalys* or meta synth* or meta-synth* or metasynth*).ti,ab,kf,hw.
16	((systematic or meta) adj2 (analys* or review)).ti,kf. or ((systematic* or quantitativ* or methodologic*) adj5 (review* or overview*)).ti,ab,kf,sh. or (quantitativ$ adj5 synthesis$).ti,ab,kf,hw.
17	(integrative research review* or research integration).tw. or scoping review?.ti,kf. or (review.ti,kf,pt. and (trials as topic or studies as topic).hw.) or (evidence adj3 review*).ti,ab,kf.
18	review.pt. and ((medline or medlars or embase or pubmed or scisearch or psychinfo or psycinfo or psychlit or psyclit or cinahl or electronic database* or bibliographic database* or computeri#ed database* or online database* or pooling or pooled or mantel haenszel or peto or dersimonian or der simonian or fixed effect or ((hand adj2 search*) or (manual* adj2 search*))).tw,hw. or (retraction of publication or retracted publication).pt.)
19	or/13-18
20	Arthroplasty, Replacement, Knee/
21	Knee Prosthesis/
22	(arthoplast$ adj3 knee$).mp. [mp=title, abstract, original title, name of substance word, subject heading word, floating sub-heading word, keyword heading word, organism supplementary concept word, protocol supplementary concept word, rare disease supplementary concept word, unique identifier, synonyms]
23	(knee$ adj3 replac$).mp. [mp=title, abstract, original title, name of substance word, subject heading word, floating sub-heading word, keyword heading word, organism supplementary concept word, protocol supplementary concept word, rare disease supplementary concept word, unique identifier, synonyms]
24	(knee$ adj3 implant$).mp. [mp=title, abstract, original title, name of substance word, subject heading word, floating sub-heading word, keyword heading word, organism supplementary concept word, protocol supplementary concept word, rare disease supplementary concept word, unique identifier, synonyms]
25	(knee$ adj3 prosthe$).mp. [mp=title, abstract, original title, name of substance word, subject heading word, floating sub-heading word, keyword heading word, organism supplementary concept word, protocol supplementary concept word, rare disease supplementary concept word, unique identifier, synonyms]
26	(knee$ adj3 endoprosthe$).mp. [mp=title, abstract, original title, name of substance word, subject heading word, floating sub-heading word, keyword heading word, organism supplementary concept word, protocol supplementary concept word, rare disease supplementary concept word, unique identifier, synonyms]
27	unicondylar.mp.
28	unicompartmental.mp.
29	or/20-28
30	12 or 19
31	29 and 30

For peer review only - http://bmjopen.bmj.com/site/about/guidelines.xhtml

Supplementary material. Economic decision models

Model structure

Potential model structures were developed by the research team with the help of surgeons, patients, and searches for previous economic models. We will start by applying a Markov decision model with tunnel states to reflect the three different time periods at risk, as depicted in Figure 1.

Figure 1. A Markov model using tunnel states to model outcomes after total knee replacement (`A').*

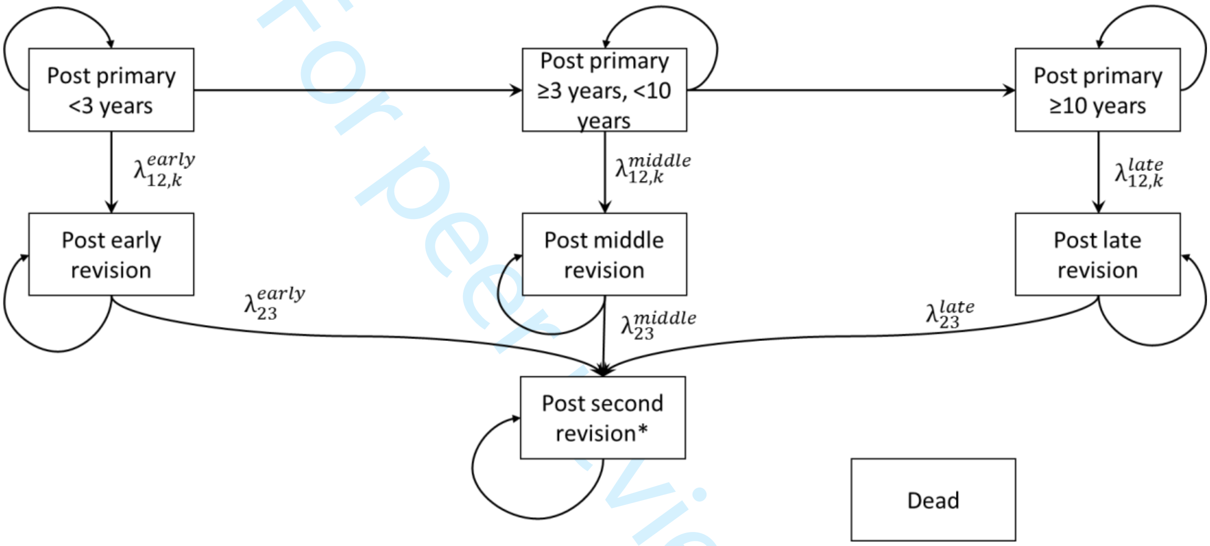

*Indices of transition rates λ_{ij} correspond to sets of states i and j: "Post TKR" ($i = 1$), Post revision ($i = 2$), and post second revision ($i = 3$).

States are divided into three groups corresponding to post primary (set 1), post revision (set 2), and post second revision (set 3) surgery. This model splits revisions into three time periods: up to 3 years post primary surgery ("early"), between 3- and 10-years post-primary ("middle"), and 10 or more years post primary surgery ("late"). This division applies to the post primary and post revision sets of states.

The three post primary states have three log hazard rates of revision $\lambda_{12,k}^{early}$, $\lambda_{12,k}^{middle}$, and $\lambda_{12,k}^{late}$, for implant k, such as:

$$\text{Equation 1. } \lambda_{12,k}^{period} = \alpha_{12}^{period} + d_{12,k}^{period}$$

In Equation 1, period is "early", "middle", or "late" first revision.

α_{12}^{period} is the log hazard of 1st revision for the baseline implant and informed by National Joint Registry (NJR) or Swedish Knee Arthroplasty Register (SKAR) data, which is constant over time within each 'period'. $d_{12,k}^{period}$ is the log hazard ratio of 1st revision for implant k

For peer review only - http://bmjopen.bmj.com/site/about/guidelines.xhtml

relative to the baseline and it is informed by the results of the network meta-analysis (NMA), combined with NJR or SKAR data. If NMA estimates are too sparse or uncertain, will use observational estimates from registries only.

These time-independent log rates are converted to annual probabilities for the 1-year cycle length using the exponential distribution

Equation 2. $P_{12,k}^{period} = 1 - \exp(-\exp(\lambda_{12,k}^{period}))$

The rates of second revision are assumed to depend on whether the first revision occurred in the "early", "middle", or "late" first revision period, with constant log hazards λ_{23}^{early}, λ_{23}^{middle}, and λ_{23}^{late}. These are again converted to probabilities using an exponential distribution

Equation 3. $P_{23}^{period} = 1 - \exp(-\exp(\lambda_{23}^{period}))$

The log rates of second revision will be estimated using NJR and SKAR data and will be assumed to be the same for all implants (hence no index k); note that they are different to the log rates of first revision.

The annual probability of death $P_M(u)$ will be assumed independent of current state or implant. It will depend on current time u as it will increase with age as in the Office of National Statistics life tables. Dependence of all log hazards of 1st and 2nd revision on age and gender will be managed through stratification into male/female and age bands (e.g. <55, 55-64, 65-74, 75+ years old).

We will also explore using an individual level continuous time semi-Markov multistate model, which simulates individual patient paths in continuous time.

A continuous model allows the hazard rate and hazard rate ratios of first revision to vary continuously, giving a log transition rate of 1st revision function $\lambda_{12,k}(u, x_i)$ that depends on current time u and, possibly, on patient characteristics x_i. The constant hazards over the early, middle, and late periods in the cohort Markov model are an approximation to this function. The log rate $\lambda_{12,k}(u, x_i)$ is generated using baseline implant hazards $\alpha_{12}(u, x_i)$ (i.e. $k = 1$) from NJR/SKAR and hazard ratios $d_{12,k}(u, x_i)$ from NJR/SKAR and the NMA for other implants k:

Equation 4. Individual patient semi-Markov : $\lambda_{12,k}(u, x_i) = \alpha_{12}(u, x_i) + d_{12,k}(u, x_i)$

Unlike the cohort Markov model the log rates of revision on the baseline implant $\alpha_{12}(u, x_i)$ and log hazard ratios $d_{12,k}(u, x_i)$ for implant k are now functions (rather than three constants).

For peer review only - http://bmjopen.bmj.com/site/about/guidelines.xhtml

The hazard of second revision $\lambda_{23}(u, t_{12,i})$ will depend on the time taken by individual patient i to experience first revision $t_{12,i}$ (i.e. time to transition to from 1st to 2nd state). Mortality is modelled as a log hazard of death $\lambda_M(u, x_i)$ which is assumed independent of current state or implant but may depend on patient characteristics x_i (e.g. age and gender)

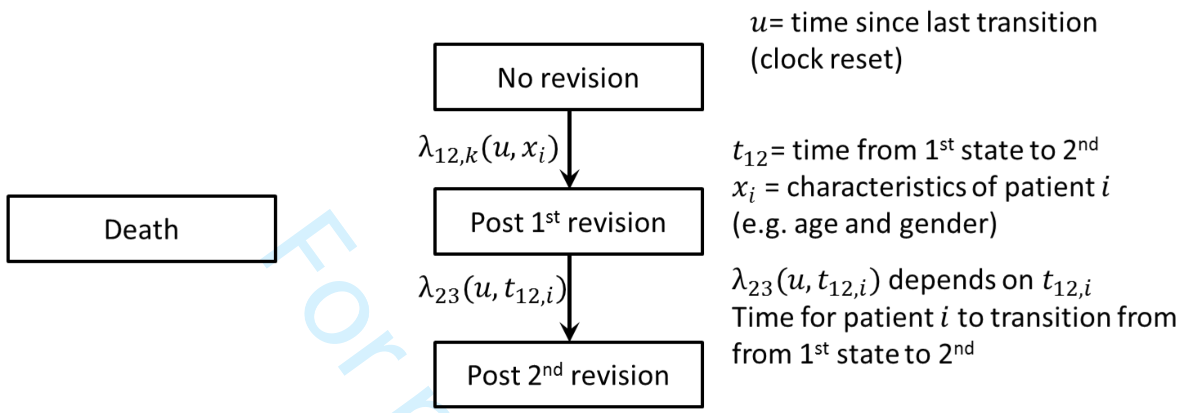

For peer review only - http://bmjopen.bmj.com/site/about/guidelines.xhtml

Supplementary material 1. PRISMA-P (Preferred Reporting Items for Systematic review and Meta-Analysis Protocols) 2015 checklist: recommended items to address in a systematic review protocol*

Section and topic	Item No	Checklist item	
**ADMINISTRATIVE INFORMATION**			
Title:			
Identification	1a	Identify the report as a protocol of a systematic review	Title
Update	1b	If the protocol is for an update of a previous systematic review, identify as such	N/A
Registration	2	If registered, provide the name of the registry (such as PROSPERO) and registration number	Provided
Authors:			
Contact	3a	Provide name, institutional affiliation, e-mail address of all protocol authors; provide physical mailing address of corresponding author	Provided
Contributions	3b	Describe contributions of protocol authors and identify the guarantor of the review	Provided
Amendments	4	If the protocol represents an amendment of a previously completed or published protocol, identify as such and list changes; otherwise, state plan for documenting important protocol amendments	N/A
Support:			
Sources	5a	Indicate sources of financial or other support for the review	NIHR funding described
Sponsor	5b	Provide name for the review funder and/or sponsor	The study is registered with the funder (the NIHR), the host (the NHS North Bristol Trust) and the sponsor (University of Bristol) and has obtained all approvals to start
Role of sponsor or funder	5c	Describe roles of funder(s), sponsor(s), and/or institution(s), if any, in developing the protocol	Described in “Funding source”
**INTRODUCTION**			
Rationale	6	Describe the rationale for the review in the context of what is already known	Introduction
Objectives	7	Provide an explicit statement of the question(s) the review will address with reference to participants, interventions, comparators, and outcomes (PICO)	Introduction

For peer review only - http://bmjopen.bmj.com/site/about/guidelines.xhtml

**METHODS**			
Eligibility criteria	8	Specify the study characteristics (such as PICO, study design, setting, time frame) and report characteristics (such as years considered, language, publication status) to be used as criteria for eligibility for the review	Methods: Eligibility criteria
Information sources	9	Describe all intended information sources (such as electronic databases, contact with study authors, trial registers or other grey literature sources) with planned dates of coverage	Methods: Eligibility criteria
Search strategy	10	Present draft of search strategy to be used for at least one electronic database, including planned limits, such that it could be repeated	Table 2
Study records:			
Data management	11a	Describe the mechanism(s) that will be used to manage records and data throughout the review	Methods: data management
Selection process	11b	State the process that will be used for selecting studies (such as two independent reviewers) through each phase of the review (that is, screening, eligibility and inclusion in meta-analysis)	Methods: data management
Data collection process	11c	Describe planned method of extracting data from reports (such as piloting forms, done independently, in duplicate), any processes for obtaining and confirming data from investigators	Methods: data management
Data items	12	List and define all variables for which data will be sought (such as PICO items, funding sources), any pre-planned data assumptions and simplifications	Methods: data management
Outcomes and prioritization	13	List and define all outcomes for which data will be sought, including prioritization of main and additional outcomes, with rationale	Introduction: objectives<br>Methods: types of outcome measures
Risk of bias in individual studies	14	Describe anticipated methods for assessing risk of bias of individual studies, including whether this will be done at the outcome or study level, or both; state how this information will be used in data synthesis	Methods: risk of bias assessment
Data synthesis	15a	Describe criteria under which study data will be quantitatively synthesised	Methods; pairwise meta-analysis<br>Methods: network meta-analysis
	15b	If data are appropriate for quantitative synthesis, describe planned summary measures, methods of handling data and methods of combining data from studies, including any planned exploration of consistency (such as I^2, Kendall's τ)	Methods; pairwise meta-analysis<br>Methods: network meta-analysis
	15c	Describe any proposed additional analyses (such as sensitivity or subgroup analyses, meta-regression)	Methods: Heterogeneity and subgroup analyses
	15d	If quantitative synthesis is not appropriate, describe the type of summary planned	Methods: pairwise meta-analysis

For peer review only - http://bmjopen.bmj.com/site/about/guidelines.xhtml

Meta-bias(es)	16	Specify any planned assessment of meta-bias(es) (such as publication bias across studies, selective reporting within studies)	Methods: risk of bias assessment<br>Methods: pairwise meta-analysis
Confidence in cumulative evidence	17	Describe how the strength of the body of evidence will be assessed (such as GRADE)	Methods: quality of evidence

*** It is strongly recommended that this checklist be read in conjunction with the PRISMA-P Explanation and Elaboration (cite when available) for important clarification on the items. Amendments to a review protocol should be tracked and dated. The copyright for PRISMA-P (including checklist) is held by the PRISMA-P Group and is distributed under a Creative Commons Attribution Licence 4.0.**

From: Shamseer L, Moher D, Clarke M, Ghersi D, Liberati A, Petticrew M, Shekelle P, Stewart L, PRISMA-P Group. Preferred reporting items for systematic review and meta-analysis protocols (PRISMA-P) 2015: elaboration and explanation. BMJ. 2015 Jan 2;349(jan02 1):g7647.

For peer review only
